# POSTN^+^ CAF-Derived Migrasomes Drive Hepatocellular Carcinoma Progression and Confer Resistance to Immunotherapy

**DOI:** 10.34133/research.0950

**Published:** 2025-10-22

**Authors:** Zhenhua Zhu, Kangnan Zhang, Wei Wu, Jingyi Zhou, Mingming Zhang, Jiong Chen, Jinlu Han, Na Wang, Biao Li, Wenhao Weng, Qinghui Zhang, Min Zhou, Ling Xu

**Affiliations:** ^1^ Department of Gastroenterology, Tongren Hospital, Shanghai Jiao Tong University School of Medicine, Shanghai 200336, China.; ^2^ Department of Clinical Laboratory, Shanghai Children’s Hospital, School of Medicine, Shanghai Jiao Tong University, Shanghai 200062, China.; ^3^ Clinical Research Institute, Shanghai General Hospital, Shanghai Jiao Tong University School of Medicine, Shanghai 200080, China.; ^4^ School of Artificial Intelligence and Data Science, University of Science and Technology of China, Hefei 230000, China.; ^5^ Department of Oncology, Shanghai General Hospital, Shanghai Jiao Tong University School of Medicine, Shanghai 200080, China.; ^6^ Shanghai Key Laboratory for Tumor Microenvironment and Inflammation, Department of Biochemistry and Molecular Cell Biology, Shanghai Jiao Tong University School of Medicine, Shanghai 200025, China.; ^7^ Dental & Ophthalmic Clinic of Putuo District, Shanghai 200336, China.; ^8^ Department of Clinical Laboratory, Tongren Hospital, Shanghai Jiao Tong University School of Medicine, Shanghai 200336, China.; ^9^ Department of Critical Care Medicine, The First Affiliated Hospital of University of Science and Technology of China, Division of Life Sciences and Medicine, University of Science and Technology of China, Hefei, Anhui Province 230031, China.

## Abstract

Cancer-associated fibroblasts (CAFs) are known to shape the tumor microenvironment, yet the role of extracellular vesicles they produce, particularly migrasomes, in hepatocellular carcinoma (HCC) remains largely unexplored. In this study, we identified a subset of POSTN^+^ CAFs as the main source of migrasomes in HCC, predominantly located at the tumor–stroma interface. Transcriptomic data from TCGA and ICGC were used to compute a Migrasome_Score via GSVA, revealing strong associations with poor prognosis and immune checkpoint blockade resistance across cancers. Mechanistically, POSTN^+^ CAF-derived migrasomes enhanced endothelial angiogenic activity by delivering VEGFA and activating the VEGFR2–PI3K–AKT–eNOS pathway, promoting vascular remodeling within tumors. Migrasomes also induced malignant reprogramming of nearby hepatocytes by boosting oxidative phosphorylation and reactive oxygen species (ROS) production, leading to DNA damage and oncogenic transformation—effects reversed by ROS scavenging. Additionally, POSTN^+^ CAFs and their migrasomes physically hindered immune cell infiltration, forming a barrier that shielded tumor cells from immune surveillance. Together, these findings identify POSTN^+^ CAF-derived migrasomes as key drivers of HCC progression by promoting angiogenesis, malignant transformation, and immune exclusion. Their strong links to poor prognosis and immunotherapy resistance suggest that they may serve as promising therapeutic targets and prognostic biomarkers in HCC and beyond.

## Introduction

Migrasomes, a newly identified class of large extracellular vesicles (EVs) enriched in tetraspanin 4 (TSPAN4), are formed during cell migration and have emerged as important mediators of intercellular communication in cancer. They are known to transport bioactive molecules—such as microRNAs, integrins, and matrix metalloproteinases—to reshape the tumor microenvironment and premetastatic niches [1–5]. In hepatocellular carcinoma (HCC), elevated migrasome levels have been associated with venous invasion and early recurrence. However, their precise cellular origin and context-specific functional roles remain poorly defined. In particular, whether migrasomes mediate immune evasion or promote malignant transformation in HCC—and how their generation is influenced by stromal heterogeneity—remains unclear. These questions are especially relevant given the spatial complexity and stromal dominance of the HCC tumor microenvironment.

HCC represents the predominant form of liver malignancy and contributes substantially to global cancer mortality [6–8]. Despite advances in immunotherapy and targeted treatments, more than 70% of patients still experience recurrence or metastasis, emphasizing the urgent need to elucidate the cellular drivers of therapeutic resistance [9–11]. Increasing evidence implicates cancer-associated fibroblasts (CAFs) as central orchestrators of the immunosuppressive and pro-metastatic tumor microenvironment [12–14]. Through paracrine signaling and extracellular matrix (ECM) remodeling, CAFs not only promote angiogenesis and metastasis but also contribute to resistance against immune checkpoint blockade (ICB) [15–19].

Recent spatial transcriptomic studies have revealed that CAFs actively cooperate with immune cells to establish localized tumor immune barriers (TIBs). Liu et al. [20] demonstrated that CAFs and SPP1^+^ macrophages engage in reciprocal signaling that excludes cytotoxic T cells from the tumor core, aided by ECM stiffening and CXCL12 secretion. Wang et al. [21] further identified a POSTN^+^ CAF subset that amplifies this immunosuppressive niche by activating macrophages and recruiting regulatory T (Treg) cells via interleukin-6 (IL-6)/signal transducer and activator of transcription 3 (STAT3) and transforming growth factor-β (TGF-β) signaling. While these studies highlight the spatial and functional complexity of CAF subsets, it remains unknown whether these same CAFs can produce migrasomes—and if so, whether migrasomes act as effectors of CAF-mediated immunosuppression, angiogenesis, or tumor progression.

Moreover, previous studies have shown that tumor-derived EVs can reprogram normal neighboring cells, altering their metabolic and transcriptional profiles [22]. EVs derived from tumor cells have been shown to enhance proliferation, induce drug resistance, disrupt endoplasmic reticulum homeostasis, and promote epithelial-to-mesenchymal transition in normal cells. In addition, tumor-derived EVs can trigger genomic instability in healthy cells, potentially leading to new mutations and malignant transformation [22,23]. Given that CAFs are key regulators of HCC progression and often localize at the invasive front—a region rich in EV trafficking—we hypothesized that CAF-derived migrasomes may represent a novel mechanism for promoting malignant transformation of adjacent hepatocytes. This process would conceptually align with the “field cancerization” model [24], wherein tumor-adjacent tissues acquire oncogenic traits before overt malignant conversion, potentially mediated by stromal-derived signals.

To test this hypothesis and map the distribution and function of CAF-derived migrasomes in the HCC microenvironment, we integrated single-cell transcriptomics, spatial transcriptomics, and multiplex immunohistochemistry. We identify POSTN^+^ CAFs as dominant migrasome producers and uncover their critical roles in promoting angiogenesis via vascular endothelial growth factor A (VEGFA)–vascular endothelial growth factor receptor 2 (VEGFR2) signaling, reprogramming neighboring hepatocytes through reactive oxygen species (ROS)-mediated oxidative phosphorylation, and creating a physical barrier that excludes cytotoxic immune cells. Spatially, these migrasomes are enriched at the tumor–stroma interface and correlate with resistance to ICB—a pattern conserved across multiple cancers. These findings redefine migrasomes as stromal-derived effectors that accelerate HCC progression and nominate the POSTN^+^ CAF–migrasome axis as a novel therapeutic vulnerability in microenvironment-driven malignancies.

## Results

### Migrasome abundance stratifies HCC progression and predicts dismal clinical outcomes

**Fig. 1.**
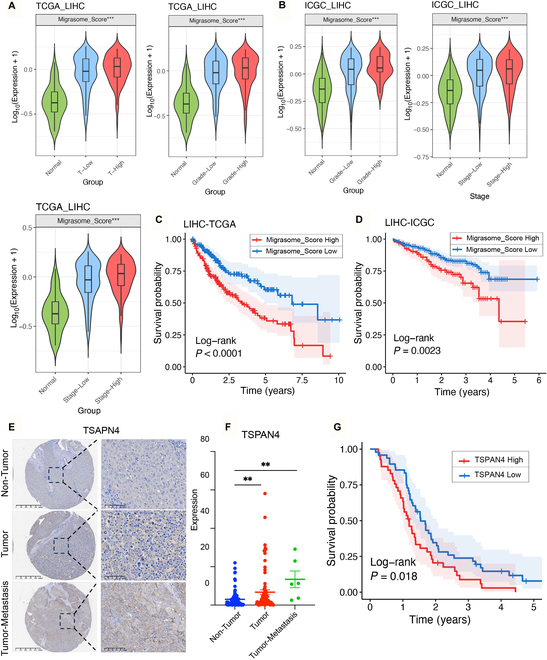
High Migrasome_Score indicates poor prognosis and tumor aggressiveness in HCC. (A and B) Differential expression analysis of Migrasome_Score across different T stages, grades, and tumor types for normal and tumor samples in the TCGA-LIHC (liver hepatocellular carcinoma) (A) and ICGC-LIRI (Liver Cancer-RIKEN) (B) datasets. (C and D) Kaplan–Meier curves showing the survival differences of Migrasome_Score between sample groups in TCGA-LIHC (C) and ICGC-LIRI (D) datasets. The blue line indicates Migrasome_Score = Low, while the red line indicates Migrasome_Score = High. (E) Immunohistochemistry results of TSPAN4 in HCC tissue microarray (TMA) samples, including 90 HCC tumor tissues and 90 adjacent nontumor tissues, with representative images shown for 2 samples (scale bars, 100 μm). (F) Statistical representation of TSPAN4 protein staining differences across tumor, nontumor, and tumor metastasis in TMA samples. (G) Survival curve of TSPAN4 in 90 clinical HCC samples. Data are expressed as mean ± SD. Two-tailed unpaired Student’s *t* tests. One-way analysis of variance (ANOVA) was used followed by Tukey’s post hoc test to determine the statistical significance. *P < 0.05; **P < 0.01; ***P < 0.001; ns, not significant.

Consistent with prior evidence implicating migrasomes in oncogenesis [25], we observed pronounced enrichment of migrasome-related gene signatures in HCC versus adjacent nontumor tissues (*P* < 0.001, Wilcoxon test). To systematically interrogate their clinical relevance, we stratified HCC samples from The Cancer Genome Atlas (TCGA) and International Cancer Genome Consortium (ICGC) cohorts by T stage, clinical stage, and histological grade, defining low (stages I to II/grades I to II) versus high (stages III to IV/grades III to IV) migrasome expression groups. A Migrasome_Score—calculated via gene set variation analysis (GSVA) using 7 core migrasome biogenesis regulators (TSPAN4, EOGT, PIGK, CERS5, CERT1, NDST1, and ITGA5) [26–28]—revealed stepwise escalation of migrasome activity across tumor progression milestones. The results revealed that in both TCGA and ICGC cohorts, migrasome expression was lowest in normal liver tissues, with the Migrasome_Score progressively increasing in parallel with tumor stage and grade (Fig. 1A and B). Expression patterns of individual genes across different stages and grades demonstrated a consistent upward trend corresponding with the Migrasome_Score (Figs. S1A to C and S2A and B). Further analysis indicated that patients in the high Migrasome_Score group exhibited poorer prognosis in both TCGA and ICGC liver cancer cohorts (Fig. 1C and D).

To validate these findings, we performed experimental analysis using liver cancer tissue microarrays (TMAs). Given that TSPAN4 is considered a classic marker of migrasomes, we selected TSPAN4 as the representative marker and categorized lymphatic and distant metastatic samples as the “metastasis group”. TMA experiments corroborated the aforementioned analysis, showing that TSPAN4 expression was lowest in noncancerous tissue, higher in nonmetastatic tumors, and highest in metastatic liver cancer samples (Fig. 1E and F). Prognostic analysis based on clinical information from the TMAs (Table S1) further revealed that patients with high TSPAN4 expression had markedly poorer prognosis (Fig. 1G). In conclusion, migrasome expression is positively correlated with the severity of HCC, and high migrasome expression is strongly associated with poor prognosis in HCC patients.

### CAFs are the predominant source of migrasomes in the HCC microenvironment

**Fig. 2.**
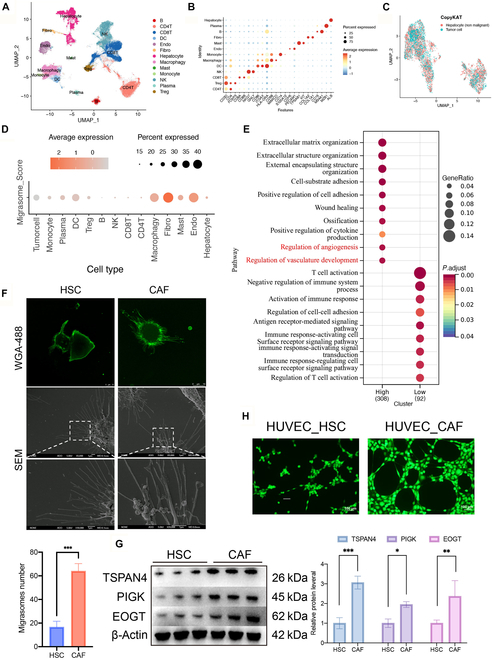
CAFs are the main migrasome source in HCC revealed by single-cell analysis. (A) The liver cancer single-cell dataset is clustered into 13 groups, each displayed in different colors. (B) Expression levels of selected known marker genes in uncategorized cells from liver cancer tissues. (C) UMAP visualization of single-cell transcriptomes following CopyKAT analysis. Cells were classified into tumor cells (aneuploid) and hepatocytes (diploid) based on large-scale copy number variation (CNV) inference from scRNA-seq data. (D) Dot plots showing the Migrasome_Score expression across 14 distinct cell clusters. (E) GO enrichment analysis bubble plot for differential genes between Fibro-Migrasome_Score High (308 DEGs) and Fibro-Migrasome_Score Low (92 DEGs) groups, where the values “308” (High) and “92” (Low) represent the counts of DEGs in each group. Bubble size indicates the GeneRatio (the ratio of the number of genes enriched in a given pathway to the total number of input genes), and the color depth represents the adjusted *P* value (*P*.adjust), with deeper red indicating higher significance. (F) Observation of HSC and CAF cells by SEM (scale bars, 1 μm). HSC and CAF cells were cultured for 12 h, stained with 1 μg/ml wheat germ agglutinin (WGA)-Alexa 488, and observed under confocal microscopy (scale bars, 10 μm). (G) Western blot (WB) analysis of migrasome marker expression in HSC and CAF cell lines (*n* = 3 samples per group). (H) Matrigel assay to determine the ability of HUVECs to form capillaries when cocultured with migrasomes collected from HSC and CAF cells (scale bars, 100 μm). Data are expressed as mean ± SD. Two-tailed unpaired Student’s *t* tests. *P < 0.05; **P < 0.01; ***P < 0.001; ns, not significant.

To deconvolve migrasome cellular origins within the HCC ecosystem, we integrated single-cell RNA sequencing (scRNA-seq) data from liver cancer samples in the GSE125449, GSE156337, GSE98638, and European Genome-phenome Archive (EGA) datasets for detailed analysis. Initially, we utilized the Harmony algorithm to perform batch correction on principal components, subsequently generating unified uniform manifold approximation and projection (UMAP) embeddings and conducting graph-based clustering analysis to annotate the cell types in each cluster. A total of 13 major cell types were identified (Fig. 2A), including CD4^+^ T cells; CD3D^+^CD4^+^FOXP3^+^ Treg cells, CD8A^+^CD8B^+^ T cells, NKG7^+^GNLY^+^ natural killer (NK) cells, CD86^+^ITGAX^+^HLA-DRA^+^ dendritic cells (DCs), CD68^+^MARCO^+^ macrophages, CD14^+^S100A12^+^ monocytes, CDH5^+^PECAM1^+^ endothelial cells, TPSAB1^+^KIT^+^ mast cells, COL1A1^+^COL1A2^+^ fibroblasts, CD19^+^MS4A1^+^ B cells, MZB1^+^ plasma cells, and ALB^+^KRT18^+^ hepatocytes (Fig. 2B). Tumor cells, derived from hepatocytes, were identified based on large-scale aneuploidy using copy number karyotyping of aneuploid tumors (CopyKAT) [29], which infers genome-wide copy number profiles from scRNA-seq data (Fig. 2C). Upon calculating the Migrasome_Score for various cell types, we observed that fibroblasts exhibited the highest migrasome expression (Fig. 2D), indicating that fibroblasts are the principal source of migrasomes in the HCC microenvironment. Given the critical role of CAFs in tumor progression, we further divided the samples into high and low migrasome expression groups based on the median Migrasome_Score in CAFs and performed Gene Ontology (GO) functional enrichment analysis. This analysis was based on 308 differentially expressed genes (DEGs) in the high migrasome expression group and 92 DEGs in the low expression group, with the high group showing marked activation of angiogenesis-related signaling pathways (Fig. 2E). Considering that hepatic stellate cells (HSCs) can transdifferentiate into CAFs and that CAFs play a pivotal role in HCC progression, we utilized immunofluorescence and scanning electron microscopy (SEM) to observe that CAFs produced substantially more migrasomes than HSCs (Fig. 2F). Additionally, Western blot (WB) analysis demonstrated that CAFs contained significantly more migrasomes than HSC cells (Fig. 2G). Subsequently, we performed angiogenesis assays by introducing migrasomes from HSCs and CAFs into human umbilical vein endothelial cells (HUVECs). The results demonstrated that CAF-derived migrasomes significantly enhanced the angiogenic potential of HUVECs (Fig. 2H). Angiogenesis is a hallmark of early cancer metastasis, with tumors typically remaining 1 to 2 mm in diameter in the absence of new blood vessels [30]. Thus, angiogenesis marks the transition of tumors from a dormant to a proliferative state. Taken together, CAFs are the predominant source of migrasomes in the HCC microenvironment, and their role in promoting HCC progression, particularly in angiogenesis, is critical.

### The role of CAF-derived migrasomes in maintaining liver cancer cell viability

Given that ischemic necrosis is frequently observed in poorly vascularized regions of HCC [31], the pro-angiogenic function of CAF-derived migrasomes may play a critical role in maintaining tumor viability by ensuring an adequate supply of nutrients and oxygen. This mechanism could help prevent necrosis and support sustained malignancy, particularly in hypoxic tumor zones where vascular remodeling is essential for tumor progression.

To further explore this hypothesis and examine how CAF-migrasomes spatially interact with tumor and vascular structures, we performed integrative spatial transcriptomic mapping of human HCC specimens.

**Fig. 3.**
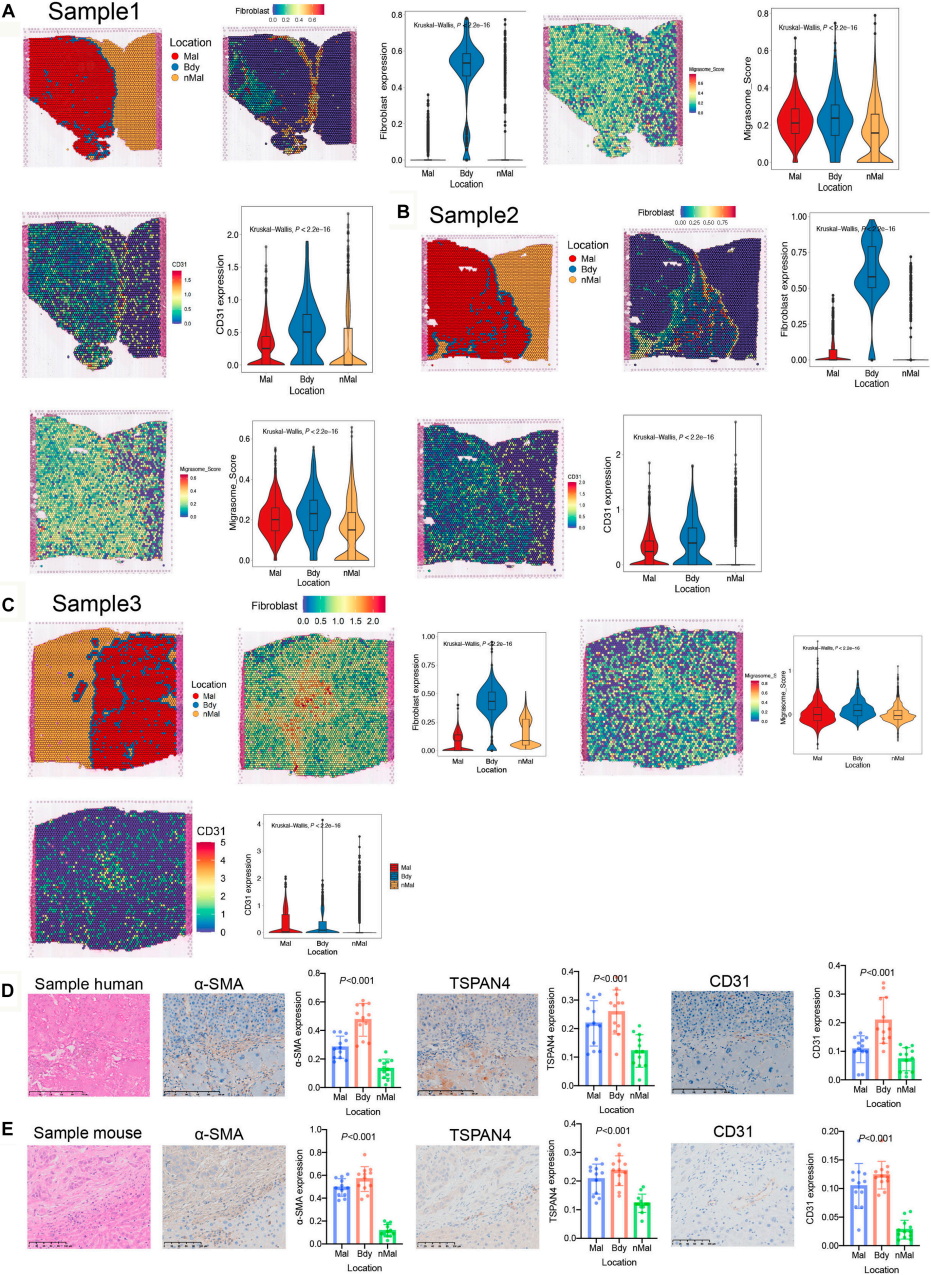
Migrasomes localize predominantly at the HCC tumor border revealed by spatial transcriptomics. (A to C) Expression and localization of fibroblast, migrasome, and CD31 in liver cancer spatial transcriptomic samples from the SpatialTME database, with statistical plots for Malignant, Boundary, and Non-Malignant regions. (D) Expression and localization of α-SMA, TSPAN4, and CD31 in human liver cancer samples, with statistical plots for Malignant, Boundary, and Non-Malignant regions (scale bars, 250 μm; *n* = 12 samples per group). (E) Expression and localization of α-SMA, TSPAN4, and CD31 in mouse liver cancer samples, with statistical plots for Malignant, Boundary, and Non-Malignant regions (scale bars, 250 μm, *n* = 12 samples per group). Mal, Malignant region; Bdy, Boundary region; nMal, Non-Malignant region. Data are expressed as mean ± SD. One-way ANOVA was used followed by Tukey’s post hoc test to determine the statistical significance. *P < 0.05; **P < 0.01; ***P < 0.001; ns, not significant.

This analysis revealed pronounced enrichment of CAFs and migrasome-associated signals (Migrasome_Score) at the tumor–stroma interface, with secondary localization in malignant regions (Fig. 3A to C). Spatial gene expression profiling confirmed elevated migrasome-related transcripts at the invasive front (Fig. S3B to D). Angiogenic hotspots, marked by CD31^+^ endothelial cell density, colocalized with migrasome-rich zones (Fig. 3A to C), aligning with prior reports of migrasome-mediated vascular remodeling.

Validation in patient-derived HCC tissues (Table S2) and murine models demonstrated conserved spatial patterning: Hematoxylin and eosin (H&E)-stained boundaries showed CAFs [α-smooth muscle actin (α-SMA^+^)] and migrasomes (TSPAN4^+^) concentrated at tumor margins, while vessels (CD31^+^) exhibited radial extension from these regions into malignant niches (Fig. 3D and E). These findings support the notion that CAF-migrasome-mediated angiogenesis not only facilitates vascular remodeling but also functionally contributes to maintaining the energy supply required for tumor persistence, especially in hypoxic and necrosis-prone regions. Collectively, these observations suggested that CAF-derived migrasomes may actively remodel the tumor microenvironment by establishing a pro-angiogenic niche supporting tumor progression.

**Fig. 4.**
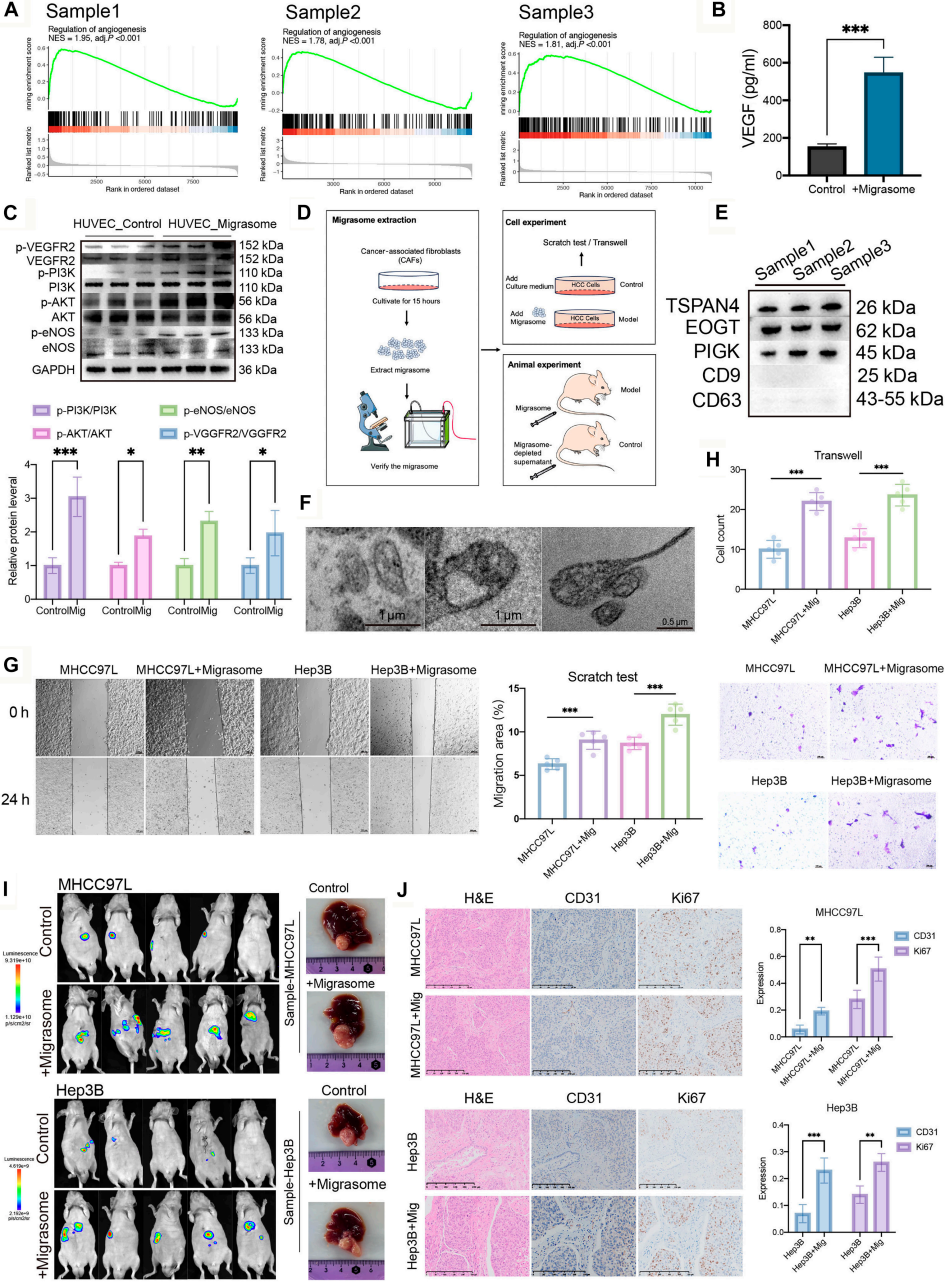
CAF-derived migrasomes activate angiogenesis-related pathways. (A) GSEA analysis of angiogenesis-related pathways in different samples. (B) ELISA experiments to detect the content of VEGF in migrasomes (*n* = 5 samples per group). (C) WB analysis of pro-angiogenic signaling in HUVECs treated with CAF-derived migrasomes or control medium without migrasomes. Quantification of protein phosphorylation in HUVECs treated with CAF-derived migrasomes or control medium without migrasomes. The levels of phosphorylated VEGFR2, PI3K, AKT, and eNOS were normalized to their respective total protein levels and are presented as p/total ratios (*n* = 3 samples per group). (D) Schematic of the study investigating the impact of CAF-derived migrasomes on liver cancer cells. (E) WB validation of isolated vesicles as migrasomes rather than exosomes. (F) Observation of enriched migrasomes by SEM (scale bars, 1 μm/0.5 μm). (G) Scratch assay investigating the wound healing capacity of liver cancer cell lines after the addition of CAF-derived migrasomes (scale bars, 100 μm; *n* = 5 samples per group). (H) Transwell assay exploring the invasion capacity of liver cancer cell lines after the addition of CAF-derived migrasomes (scale bars, 100 μm; *n* = 5 samples per group). (I) In vivo mouse model of liver cancer using MHCC97-L and Hep3B liver cancer cell lines. Mice were allowed to grow for 3 months, followed by intraperitoneal injection of d-luciferin to quantify tumor burden. Tumor sizes in vivo are represented by color gradients. Mice were then euthanized, and livers were excised and photographed. (J) H&E staining of mouse liver tissue sections and immunohistochemistry of α-SMA and Ki67, with statistical expression analysis (scale bars, 250 μm; *n* = 5 samples per group). Data are expressed as mean ± SD. Two-tailed unpaired Student’s *t* tests. *P < 0.05; **P < 0.01; ***P < 0.001; ns, not significant.

To investigate the molecular underpinnings of this spatial enrichment, we leveraged spatial transcriptomic datasets to assess the relationship between migrasome activity and angiogenesis-related signaling. In these spatial transcriptomic samples, the Bdy region showed the highest Migrasome_Score expression and was thus defined as the Migrasome High area, while the Mal region exhibited slightly lower Migrasome_Score levels and was designated the Migrasome Low area. GSEA (Gene Set Enrichment Analysis) analysis comparing these 2 zones revealed significant enrichment of the “regulation of angiogenesis” pathway across all samples. These results extend our spatial findings by indicating that migrasome accumulation may promote angiogenic signaling within malignant regions (Fig. 4A).

Based on these transcriptional signatures and their spatial colocalization with the vasculature, we hypothesized that CAF-derived migrasomes may promote angiogenesis. To identify the responsible mediators, we quantified a panel of candidate angiogenic factors (VEGFA, IL-8, ANG-2, FGF-2, CXCL12, and PDGF) in CAF-derived migrasomes by enzyme-linked immunosorbent assay (ELISA); VEGFA was the most abundant (Fig. 2G and Fig. S4A). We then performed functional blocking experiments targeting the receptors of the top 3 angiogenic factors. Specifically, VEGFA–VEGFR, IL-8–CXCR1/2, and ANG-2–Tie2 interactions were inhibited in HUVECs treated with CAF-migrasomes (Fig. S4B). Blockade of VEGFR markedly suppressed the formation of capillary-like structures, whereas inhibition of CXCR1/2 or Tie2 did not prevent angiogenesis. Together, these data indicate that VEGFA is the predominant mediator of the pro-angiogenic activity of CAF-derived migrasomes.

To directly test this hypothesis, we treated HUVECs with purified CAF-migrasomes and performed WB analysis for components of the VEGF signaling pathway. Migrasome-treated HUVECs showed increased phosphorylation of VEGFR2, phosphatidylinositol 3-kinase (PI3K), AKT, and eNOS (endothelial nitric oxide synthase), indicating that the VEGFA cargo within migrasomes is functionally active and capable of activating the VEGF–VEGFR2–PI3K–AKT–eNOS axis in endothelial cells (Fig. 4C). These molecular data provide mechanistic evidence that CAF-derived migrasomes drive tumor vascular remodeling through canonical pro-angiogenic signaling.

CAFs are well-recognized as key regulators of HCC progression, primarily through remodeling the ECM, modulating immune responses, and secreting various paracrine factors that enhance tumor cell proliferation, migration, and invasion [32]. However, the specific mechanisms by which CAFs communicate with tumor cells remain incompletely understood.

Migrasomes, a novel class of EVs abundantly produced by CAFs as demonstrated here, represent a potential new mode of intercellular communication within the tumor microenvironment. Given their spatial colocalization with malignant regions and established role in promoting angiogenesis, we hypothesized that CAF-derived migrasomes might also directly influence HCC tumor cell behavior. To test this, we investigated the effects of purified CAF-derived migrasomes on HCC cell migration, invasion, and proliferation (Fig. 4D). Migrasomes were isolated via differential ultracentrifugation and verified by transmission electron microscopy (TEM) and immunoblotting for TSPAN4 and other migrasome-specific markers (Fig. 4E and F). Treatment of low-metastatic HCC cell lines (Hep3B and MHCC97-L) with purified CAF-migrasomes induced marked pro-malignant remodeling: Transwell assays showed significant increases in migratory capacity (Hep3B: 8.68% ± 0.63% versus 11.98% ± 1.09%; MHCC97-L: 6.30% ± 0.55% versus 9.04% ± 0.93%; Fig. 4G), and Matrigel invasion assays demonstrated nearly doubled invasive potential (Hep3B: 13 ± 2 versus 24 ± 2; MHCC97-L: 10 ± 2 versus 22 ± 2; Fig. 4H). These results confirm that CAF-migrasomes exert pro-invasive and pro-migratory effects on HCC cells.

To validate these findings in vivo, we evaluated tumor growth and vascularization in mice injected with CAF-migrasomes. Tumors in the treatment group exhibited larger volumes compared to controls (Fig. 4I), along with significantly elevated expression of CD31 and Ki67 in tumor tissues (Fig. 4J), indicating enhanced angiogenesis and proliferation. Together, these findings indicate that CAF-derived migrasomes not only maintain tumor viability through enhanced angiogenesis but also promote tumor progression by directly increasing cancer cell aggressiveness.

### CAF-derived migrasomes drive malignant reprogramming of adjacent hepatocytes

Extensive evidence has demonstrated that EVs secreted by tumor cells can reprogram surrounding normal cells by altering their metabolism, morphology, and behavior—changes that are closely associated with early malignant transformation [23,33]. Given that CAFs are key stromal components actively contributing to HCC progression, we hypothesized that CAF-derived EVs, particularly migrasomes, may exert similar reprogramming effects on neighboring nonmalignant hepatocytes. This prompted us to investigate their potential role in initiating malignant traits in normal liver cells.

**Fig. 5.**
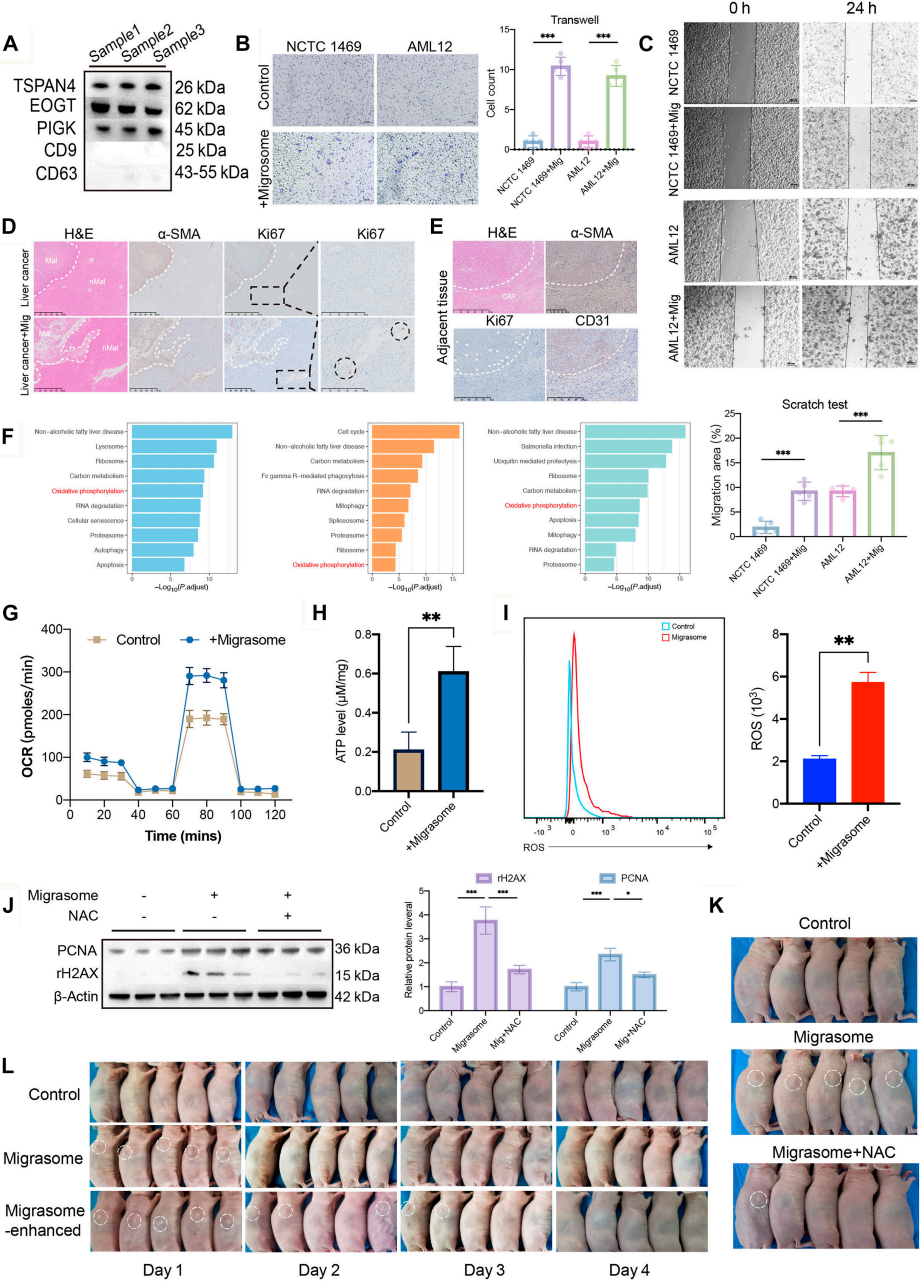
CAF-derived migrasomes induce malignant reprogramming of adjacent normal hepatocytes. (A) WB validation of isolated vesicles as migrasomes rather than exosomes. (B) Transwell assay exploring the invasion capacity of normal liver cell lines after the addition of CAF-derived migrasomes (scale bars, 100 μm; *n* = 5 samples per group). (C) Scratch assay investigating the wound healing capacity of normal liver cell lines after the addition of CAF-derived migrasomes (scale bars, 100 μm; *n* = 5 samples per group). (D) H&E staining and immunohistochemistry of α-SMA and Ki67 in tissue sections from mice with primary liver tumors, with statistical expression analysis. Nonmalignant areas (nMal) exhibiting Ki67 expression are indicated by circles (scale bars, 500 μm). (E) H&E staining and immunohistochemistry of α-SMA, CD31, and Ki67 in tissue sections from human liver cancer adjacent tissues (scale bars, 500 μm). (F) KEGG pathway enrichment analysis of spatial transcriptomic data comparing the boundary (Bdy) region and nonmalignant (nMal) region, highlighting the top 10 pathways ranked by *P* value. (G) Oxygen consumption rate (OCR) measurements in AML12 cells (Control group) and AML12 cells treated with migrasomes (+Migrasome group) (*n* = 3). (H) Intracellular ATP levels in AML12 cells (Control group) and AML12 + Migrasome cells (+Migrasome group) (*n* = 3 samples per group). (I) Flow cytometric analysis of reactive oxygen species (ROS) production in AML12 (Control) and AML12 + Migrasome (+Migrasome) cells (*n* = 3). (J) WB analysis of γH2AX and PCNA in AML12 cells (*n* = 3 samples per group). (K) Subcutaneous tumorigenicity assay in mice. Control group received AML12 cells with vehicle. Migrasome group received AML12 cells pretreated with CAF-derived migrasomes. Migrasome + NAC group received AML12 cells treated with migrasomes and NAC (*n* = 5 samples per group). (L) Subcutaneous tumorigenicity of AML12 cells treated with CAF-derived migrasomes. Control group received AML12 cells with vehicle. Migrasome group received AML12 cells pretreated with migrasomes. Migrasome-enhanced group received the same cells and an additional peritumoral migrasome injection on day 1 (*n* = 5 samples per group). Data are expressed as mean ± SD. Two-tailed unpaired Student’s *t* tests. *P < 0.05; **P < 0.01; ***P < 0.001; ns, not significant.

Having established that CAF-derived migrasomes sustain HCC aggressiveness through pro-angiogenic signaling, we interrogated their capacity to modulate neighboring nonmalignant liver cells. Migrasomes isolated from CAF-conditioned media (confirmed by immunoblotting for canonical migrasome markers; Fig. 5A) were applied to murine normal hepatocyte lines NCTC 1469 and AML12 (selected to circumvent potential HeLa contamination in human primary hepatocytes). Functional assays revealed that migrasome uptake triggered profound phenotypic remodeling: Both lines exhibited a 10-fold increase in Matrigel penetration (NCTC 1469: 1 ± 1 to 10 ± 1; AML12: 1 ± 1 to 9 ± 1; Fig. 5B) and a 2.5-fold enhancement in migratory capacity (NCTC 1469: 1.90% ± 1.11% to 9.24% ± 1.67%; AML12: 9.22% ± 0.97% to 17.06% ± 3.10%; Fig. 5C), adopting invasion-competent morphologies reminiscent of early malignant transformation.

To further validate these results, we transplanted subcutaneous tumors from mice into the liver to form orthotopic tumors. The control group was injected with phosphate-buffered saline (PBS) (Liver cancer group), while the experimental group received CAF-derived migrasomes (Liver cancer + Mig group). H&E staining revealed that the orthotopic tumors formed after subcutaneous tumor transplantation into the liver should theoretically present a clear boundary, similar to the control group. However, in the migrasome injection group, the tumor boundary was irregular. Intriguingly, Ki67 immunohistochemistry revealed sporadic proliferative clusters within peritumoral “nMal” regions of migrasome-treated mice, suggesting migrasome-induced premalignant activation (Fig. 5D).

Additionally, immunohistochemical analysis of “Adjacent tissue” from liver cancer patients showed higher CD31 expression in regions where CAFs were located, suggesting rich angiogenesis. Furthermore, Ki67 expression was significantly elevated in the “nMal” region adjacent to the CAFs (Fig. 5E). These data collectively establish that CAF-derived migrasomes not only fuel angiogenesis but also instigate malignant reprogramming of adjacent hepatocytes, effectively expanding the oncogenic field.

To further explore the mechanism by which CAF-derived migrasomes reprogram normal hepatocytes toward malignant transformation, we analyzed spatial transcriptomic datasets. Given that the Bdy region exhibited the highest Migrasome_Score across samples, we defined it as the Migrasome High area, while the nMal region, with the lowest score, was designated the Migrasome Low area. Kyoto Encyclopedia of Genes and Genomes (KEGG) pathway analysis between these regions revealed consistent and significant enrichment of the “Oxidative phosphorylation” pathway among the top 10 ranked pathways across all spatial samples (Fig. 5F). This suggests that migrasome-mediated metabolic reprogramming may involve enhancement of oxidative phosphorylation, a hallmark shift associated with increased mitochondrial activity during tumor progression.

To experimentally validate this hypothesis, we isolated migrasomes from CAFs and incubated them with normal mouse hepatocyte AML12 cells. Seahorse metabolic flux analysis revealed a significant increase in the oxygen consumption rate (OCR) following migrasome treatment, indicating enhanced mitochondrial oxidative phosphorylation (Fig. 5G). Consistently, migrasome-treated cells exhibited elevated intracellular adenosine triphosphate (ATP) levels (Fig. 5H), further supporting the activation of mitochondrial respiratory function. Concurrently, both flow cytometry and immunofluorescence analyses demonstrated a marked increase in mitochondrial ROS production (Fig. 5I and Fig. S6A), suggesting that enhanced oxidative metabolism is associated with elevated oxidative stress. This ROS accumulation may contribute to early oncogenic events by promoting DNA damage, proliferative signaling, and redox imbalance.

To further validate that CAF-derived migrasomes induce early oncogenic reprogramming of normal hepatocytes via ROS accumulation, we performed WB analysis of key markers associated with DNA damage and proliferation. Compared to the control [negative control (NC)] group, AML12 cells treated with CAF-migrasomes (Mig group) exhibited markedly elevated levels of γH2AX and PCNA, indicating increased DNA damage and proliferative activity (Fig. 5J). Notably, pretreatment with the ROS scavenger N-acetylcysteine (3 mM, Mig + NAC group) effectively reversed these molecular changes, underscoring the pivotal role of ROS in mediating migrasome-induced malignant reprogramming.

To further confirm this in vivo, we injected AML12 cells subjected to different treatments subcutaneously into immunodeficient nude mice. On day 2 post-injection, mice receiving untreated AML12 cells (Control group) showed no evidence of tumor formation. In contrast, all mice injected with migrasome-treated AML12 cells (Migrasome group) developed visible subcutaneous nodules. However, in the group where migrasomes were pretreated with 3 mM NAC prior to incubation with AML12 cells (Migrasome + NAC group), only one mouse developed a detectable subcutaneous nodule. These results further support the role of ROS as a key mediator of the malignant transformation process induced by CAF-derived migrasomes (Fig. 5K).

Next, to explore whether the malignant transformation of AML12 cells by migrasomes is dose-dependent, we designed a booster injection experiment in vivo (Fig. 5L). Mice injected with control AML12 cells (Control group) showed no evidence of tumor formation throughout the observation period. In contrast, the Migrasome group (AML12 cells pretreated with CAF-derived migrasomes) and the Migrasome-enhanced group (same pretreatment followed by a second migrasome injection) developed small palpable subcutaneous nodules as early as day 1 post-injection. Notably, on day 1, we administered a booster injection of CAF-derived migrasomes around the tumor site in mice from the Migrasome-enhanced group, whereas the other 2 groups received vehicle control.

By day 2, no visible nodules were observed in the Control group, and subcutaneous masses in the Migrasome group had begun to regress, becoming barely detectable. In contrast, tumors in the Migrasome-enhanced group showed only partial regression, with some mice still exhibiting visible subcutaneous nodules.

On day 3, neither the Control nor the Migrasome group showed any signs of tumor persistence, while 2 mice in the Migrasome-enhanced group still retained palpable subcutaneous masses. By day 4, subcutaneous tumors had completely regressed in all groups.

These findings suggest that a single exposure to CAF-derived migrasomes can transiently confer tumor-like growth capacity to normal hepatocytes, while repeated or sustained exposure enhances this effect, delaying tumor regression. Collectively, these results support the role of CAF-derived migrasomes in initiating early malignant transformation in vivo by promoting metabolic reprogramming and ROS-mediated stress in adjacent hepatocytes.

### POSTN^+^ CAFs play a pivotal role in liver cancer progression

**Fig. 6.**
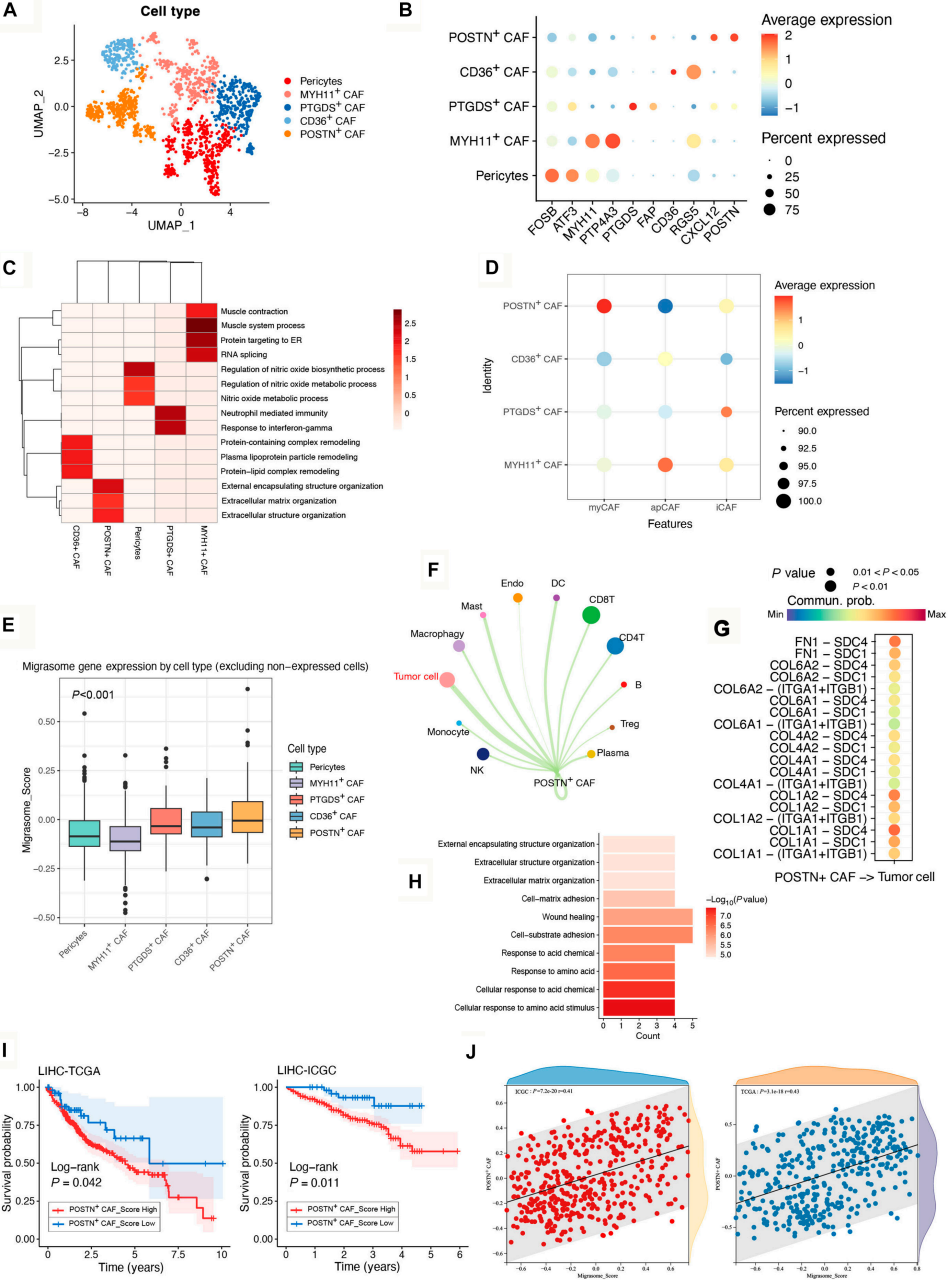
POSTN^+^ CAFs are the main migrasome producers among CAF subtypes. (A) CAF cells are divided into 5 subgroups, each displayed in different colors. (B) Expression levels of known marker genes in different CAF subtypes. (C) Functional enrichment heatmap for different CAF subtypes. (D) Bubble plot showing the average expression of 3 CAF subtypes in reclassified CAF subtypes. (E) Box plot showing Migrasome_Score expression levels in different CAF subtypes. (F) Circular plot visualizing cell communication between Endo cells, CD4 T cells, CD8 T cells, DC, hepatocyte, macrophage, monocyte, NK cells, Treg cells, mast cells, tumor cells, and POSTN^+^CAF cells. The size of the circle is proportional to the number of cells in each population, and line thickness represents the strength of interactions. (G) All important ligand–receptor pairs from POSTN^+^CAF cells to tumor cells in liver cancer tissues. Point color and size represent communication probability and *P* value. (H) Ligand–receptor functional enrichment heatmap. (I) Kaplan–Meier curve showing the impact of POSTN^+^CAF_Score on survival differences between samples in TCGA-LIHC and ICGC-LIRI datasets. The blue line indicates POSTN^+^CAF_Score = Low, while the red line indicates POSTN^+^CAF_Score = High. (J) Correlation analysis showing the relationship between POSTN^+^CAF_Score and Migrasome_Score in TCGA-LIHC and ICGC-LIRI datasets.

To further investigate the central role of CAFs in the formation of the liver cancer microenvironment, we classified the CAFs into 5 subgroups based on their gene expression profiles: POSTN^+^ CAF, CD36^+^ CAF, PTGDS^+^ CAF, MYH11^+^ CAF, and Pericytes (Fig. 6A and B). To elucidate the functional roles of these subgroups, we conducted functional enrichment analysis based on the highly expressed genes in each subgroup (Table S3) (Fig. 6C). Our findings showed that POSTN^+^ CAFs were primarily associated with ECM-related functions, such as “external encapsulating structure organization”, “extracellular matrix organization”, and “extracellular structure organization”. CD36^+^ CAFs, in contrast, were mainly associated with lipid metabolism pathways, including “protein-containing complex remodeling”, “plasma lipoprotein particle remodeling”, and “protein-lipid complex remodeling”, and PTGDS^+^ CAFs were primarily enriched in immune-related pathways, such as “neutrophil-mediated immunity” and “response to interferon-gamma”. MYH11^+^ CAFs were mainly associated with muscle and vascular functions, including “muscle contraction”, “muscle system process protein”, and “targeting to ER”. Pericytes were primarily associated with the “regulation of nitric oxide biosynthetic process”, “regulation of nitric oxide metabolic process”, and “nitric oxide metabolic process”.

To investigate the functional heterogeneity of CAFs in the tumor microenvironment, we calculated signature scores for 3 canonical subtypes: myCAFs, iCAFs, and apCAFs, based on established marker genes [34]. POSTN^+^ CAFs were primarily enriched in clusters with high myCAF and iCAF signature scores, reflecting both ECM-related and inflammatory characteristics. In contrast, PTGDS^+^ CAFs predominantly exhibited iCAF features, while CD36^+^ and MYH11^+^ CAFs aligned with the apCAF phenotype (Fig. 6D). Among all CAF subsets, POSTN^+^ CAFs exhibited the highest Migrasome_Score (Fig. 6E), suggesting their dominant role in migrasome generation. Immunofluorescence analysis further confirmed that POSTN^+^ CAFs, coexpressing α-SMA and POSTN, were spatially enriched at the tumor boundary region in human liver cancer tissues (Fig. S3A).

We also analyzed the interactions between POSTN^+^ CAFs and other cell types in the liver cancer microenvironment. The results showed that the interaction between POSTN^+^ CAFs and tumor cells was the most significant (Fig. 6F). Ligand–receptor analysis revealed that the primary interactions between POSTN^+^ CAFs and tumor cells occurred through COL1A1-SDC4, COL1A2-SDC4, and FN1-SDC4 (Fig. 6G). Functional enrichment of the ligands and receptors between POSTN^+^ CAFs and tumor cells revealed that the enriched pathways were mainly ECM-related (Fig. 6H). Based on the high-expressed genes in POSTN^+^ CAFs (*P* < 0.05), we calculated the “POSTN^+^ CAF_Score” using GSVA in both the TCGA and ICGC databases and performed survival analysis. The results demonstrated that samples with high POSTN^+^ CAF expression exhibited lower survival rates (Fig. 6I). Furthermore, we calculated the “Score” for high-expressed genes of other subtypes and performed correlation analysis with the Migrasome_Score. The results showed that only POSTN^+^ CAFs exhibited a strong correlation with the Migrasome_Score (*r* > 0.4) in both the TCGA and ICGC databases (Fig. 6J), while the correlation with other subtypes was relatively weak (Fig. S5A and B).

We next focused on POSTN^+^ CAFs to experimentally validate their role in migrasome generation and pro-tumor functions. We generated POSTN^+^ CAFs by forced overexpression of POSTN in CAFs (denoted POSTN^+^CAF); TSPAN4—a key gene in migrasome biogenesis—was subsequently silenced in these cells to produce POSTN^+^CAF_TSPAN4sh. WB showed that POSTN^+^ CAFs expressed higher levels of migrasome-associated proteins compared with bulk CAFs, whereas TSPAN4 knockdown markedly reduced these markers (Fig. S7A). Confocal immunofluorescence confirmed that POSTN^+^ CAFs produced abundant migrasomes, while TSPAN4 silencing significantly suppressed their formation (Fig. S7B), validating the effective inhibition of migrasome production.

To assess the functional consequences, migrasomes were obtained from POSTN^+^CAF_Mock cells—POSTN^+^ CAFs transfected with an empty vector to control for any effects of the transfection procedure—and from POSTN^+^CAF_TSPAN4sh cells. Invasion and migration assays demonstrated that POSTN^+^CAF_Mock-derived migrasomes significantly enhanced the invasive (Fig. S7C) and migratory (Fig. S7D) capabilities of low-aggressiveness liver cancer cell lines MHCC97L and Hep3B compared with migrasomes from TSPAN4-silenced cells. In vivo subcutaneous xenograft experiments further revealed that POSTN^+^CAF_Mock-derived migrasomes promoted larger tumor formation from MHCC97L cells (Fig. S7E).

Moreover, when normal liver cells were treated with migrasomes from POSTN^+^CAF_Mock or POSTN^+^CAF_TSPAN4sh cells, migrasomes from POSTN^+^CAF_Mock induced malignant phenotypes more effectively, whereas migrasomes from POSTN^+^CAF_TSPAN4sh displayed a reduced tumorigenic capacity in vivo, yielding fewer and less persistent subcutaneous tumors (Fig. S7F).

Collectively, these results demonstrate that POSTN^+^ CAFs are the major source of migrasomes within the CAF population, playing a pivotal role in liver cancer progression by enhancing malignant traits in liver cancer cells and promoting the transformation of normal liver cells toward a cancerous phenotype.

### POSTN^+^ CAFs and their migrasomes are correlated with immune checkpoint inhibition treatment outcomes

**Fig. 7.**
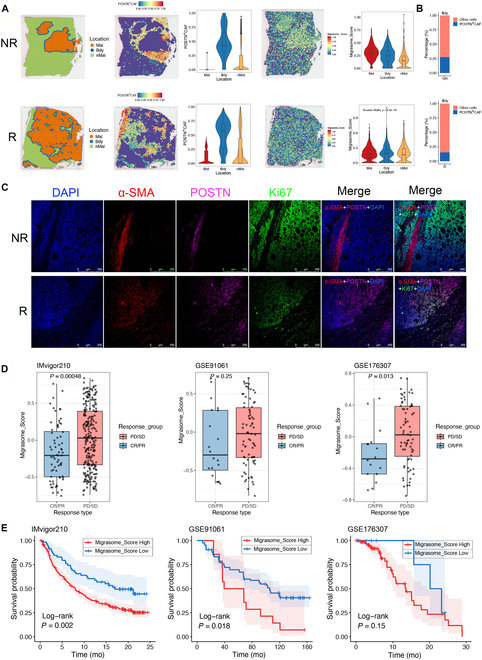
Migrasomes impair immunotherapy efficacy. (A) Spatial expression and localization of POSTN^+^CAF, migrasomes, and Migrasome_Score in liver cancer spatial transcriptomic samples, with statistical plots for Malignant, Boundary, and Non-Malignant regions. (B) Percentage of POSTN^+^CAF cells in the Boundary region. (C) Immunofluorescence staining of human liver cancer samples, with red representing α-SMA (CAF marker), magenta representing POSTN (POSTN^+^CAF marker), green representing Ki67 (liver cancer cell proliferation), and blue representing 4′,6-diamidino-2-phenylindole (DAPI) (cell nuclei) (scale bars, 500 μm). (D) Box plot comparing Migrasome_Score expression in treatment-responsive and nonresponsive samples from the immunotherapy datasets IMvigor210, GSE91061, and GSE176307. (E) Kaplan–Meier curve showing the survival differences of Migrasome_Score in IMvigor210, GSE91061, and GSE176307 datasets. The blue line indicates Migrasome_Score = Low, while the red line indicates Migrasome_Score = High. Data are expressed as mean ± SD. Two-tailed unpaired Student’s *t* tests. One-way ANOVA was used followed by Tukey’s post hoc test to determine the statistical significance. *P < 0.05; **P < 0.01; ***P < 0.001; ns, not significant.

Spatiotemporal mapping of ICB-treated HCC samples revealed that POSTN^+^ CAFs and their migrasomes predominantly localized to the “Bdy” region, forming a cage-like structural niche that physically segregated “Mal” and adjacent nonmalignant “nMal” compartments (Fig. 7A). To explore differences between responders and nonresponders to ICB therapy, we quantified the proportion of POSTN^+^ CAFs in the “Bdy” region (Fig. 7B). Consistent with our previous findings [35], nonresponder samples exhibited a significantly higher abundance of POSTN^+^ CAFs in this region. Furthermore, these samples showed elevated Migrasome_Scores within malignant areas. These observations suggest that the spatial enrichment of POSTN^+^ CAFs may contribute to the establishment of a migrasome-enriched niche that is associated with reduced immune cell infiltration into the tumor core, although direct mechanistic evidence remains to be established.

To validate this hypothesis, we conducted immunohistochemical staining on liver cancer samples from both nonresponder and responder groups following ICB treatment (Fig. 7C). The results showed that nonresponder samples had significantly higher numbers of POSTN^+^ CAFs compared to responder samples, with Ki67 expression being markedly elevated in nonresponders. In contrast, the number of CAFs and POSTN^+^ CAFs was notably lower in responder samples. These findings are consistent with the idea that POSTN^+^ CAFs and their migrasomes may contribute to shaping an “immune barrier” that influences ICB efficacy, and are in line with the observations by Liu et al. [20] and Wang et al. [21], who also reported that POSTN^+^ CAFs preferentially localize at the tumor boundary where they may exert a physical barrier function against immune infiltration.

In the TCGA and ICGC liver cancer cohorts, we next calculated the Migrasome_Score, Immune Suppression_Score [36], and a POSTN^+^CAF_Score derived from the top 20 highly expressed genes in the POSTN^+^ CAF subpopulation. Migrasome_Score was significantly correlated with Immune Suppression_Score (TCGA: *R* = 0.44, *P* < 0.0001; ICGC: *R* = 0.37, *P* < 0.0001) (Fig. S8A). Similarly, POSTN^+^CAF_Score showed a strong positive correlation with Immune Suppression_Score (TCGA: *R* = 0.43, *P* < 0.0001; ICGC: *R* = 0.62, *P* < 0.0001) (Fig. S8A). At the single-cell level, POSTN^+^ CAFs exhibited higher immune suppression scores than other CAF subsets (Fig. S8B), and GSEA further confirmed their enrichment in multiple immune-suppressive and ECM-associated pathways (Fig. S8C).

To provide functional evidence, we isolated POSTN^+^ CAF-derived migrasomes and injected them intravenously into mice bearing orthotopic liver tumors (administered once every 2 d, followed by analysis 1 week later). Tumor tissues were dissociated into single-cell suspensions and analyzed by flow cytometry. Cell viability was assessed using L/D dye, leukocytes were identified by CD45, T cells were identified by CD3, cytotoxic T cells were identified by CD8, and exhausted T cells were defined as PD1^+^TIM3^+^ subsets. Compared with control mice (Liver cancer_Control group), the Liver cancer_Migrasome group exhibited markedly higher proportions of dysfunctional and exhausted T cells (Fig. S8D). These results indicate that POSTN^+^ CAF-derived migrasomes have immunosuppressive potential in vivo.

To assess the generalizability of these findings, we analyzed ICB treatment datasets from Bladder Cancer (IMvigor210), Melanoma (GSE91061), and Metastatic Urothelial Carcinoma (GSE176307). The results indicated that in samples with poor immune treatment responses, such as stable disease (SD) and progressive disease (PD), migrasome expression was higher. In contrast, samples with better responses, such as partial response (PR) and complete response (CR), exhibited lower migrasome expression (Fig. 7D). Survival analysis further revealed that patients with high migrasome expression had poorer prognosis (Fig. 7E). These findings suggest that the expression levels of migrasomes are closely associated with cancer patient responses to ICB therapy and their prognosis.

In summary, the results of this section indicate that in HCC, POSTN^+^ CAFs and their migrasomes are predominantly enriched in the “Bdy” region and are closely associated with T cell exhaustion and immune suppression. These findings suggest that POSTN^+^ CAFs and their migrasomes may serve as key determinants for predicting responses to immunotherapy and as potential targets for therapeutic intervention.

## Discussion

Despite substantial advances in understanding the progression and treatment of liver cancer, current therapeutic strategies remain inadequate for many patients, and several key molecular mechanisms remain unclear [37,38]. Previous studies have shown that migrasome expression is closely associated with liver cancer metastasis [25]. In this study, by integrating TCGA and ICGC datasets, we found that the Migrasome_Score increases progressively with tumor stage (T stage), clinical stage, and pathological grade. This trend correlates with poor patient prognosis, supporting a central role for migrasomes in promoting liver cancer progression.

A major focus of our work was identifying the cellular origin of migrasomes within the liver cancer microenvironment. ScRNA-seq revealed that CAFs are the predominant source of migrasomes in liver cancer. This finding underscores the crucial role of CAFs in modulating tumor behavior. Functional analyses further demonstrated that CAF-derived migrasomes activate pro-angiogenic signaling pathways, thereby facilitating tumor vascularization and supporting cancer cell proliferation and dissemination—findings that align with previous studies on CAF-mediated angiogenesis [39–41].

Spatial transcriptomic data confirmed that CAFs are primarily located at the tumor boundary, a distribution pattern also reported by Liu et al. [20] using spatial transcriptomics and multiplexed single-cell profiling. Our analysis further revealed that CAF-derived migrasomes are enriched at the tumor–stroma boundary. These migrasomes exhibit bidirectional activity that contributes to HCC progression. Inwardly, they diffuse into the tumor core, where they promote angiogenesis by delivering VEGFA and activating the VEGFR2–PI3K–AKT–eNOS signaling axis. This facilitates the formation of a dense vascular network that supplies oxygen and nutrients, supporting sustained tumor proliferation and invasion. Outwardly, CAF-derived migrasomes interact with adjacent nonmalignant hepatocytes, enhancing mitochondrial oxidative phosphorylation and triggering the accumulation of ROS. This redox imbalance triggers ROS-mediated DNA damage responses, promoting early oncogenic reprogramming and expansion of the malignant field. Together, these functions suggest that CAF-derived migrasomes not only reinforce the growth and aggressiveness of established tumor cells but also remodel the surrounding microenvironment to favor malignant transformation and disease progression.

The advancement of single-cell transcriptomic technologies has enabled the detailed classification of CAF subpopulations in the tumor microenvironment [42,43]. Based on established biomarkers [14], our study identified 5 CAF subsets: POSTN^+^ CAFs, CD36^+^ CAFs, PTGDS^+^ CAFs, MYH11^+^ CAFs, and pericytes. Among these, POSTN^+^ CAFs were found to secrete the highest levels of migrasomes. POSTN (periostin), a matricellular protein previously linked to poor prognosis in multiple cancer types—including non-small cell lung [44], gastric [45], ovarian [46], thyroid [47], breast [48], and esophageal [49] cancers—was also strongly associated with liver cancer progression in our analysis. Notably, POSTN^+^ CAFs were also localized predominantly at the tumor boundary, consistent with earlier findings [21].

In addition to their biochemical roles, POSTN^+^ CAFs may influence the mechanical properties of the tumor microenvironment. It is well established that POSTN is a key matricellular protein involved in ECM remodeling and tissue stiffening, which are hallmarks of fibrotic and tumor-associated stroma [50]. Increased ECM stiffness has been shown to elevate cellular membrane tension and actomyosin contractility [51,52], both of which are known to promote migrasome biogenesis. Thus, POSTN^+^ CAFs may facilitate migrasome production not only through their secretory activity but also by mechanically reprogramming the tumor–stroma interface. This dual regulation—via biochemical and biomechanical mechanisms—could synergistically enhance migrasome formation and dissemination. These insights suggest a potential mechanotransduction link between CAF-induced ECM stiffening and migrasome-mediated malignant reprogramming of adjacent hepatocytes. To experimentally explore this hypothesis, we cultured CAFs on VitroGel hydrogels with varying stiffness to mimic the mechanical changes in liver cancer progression. We found that increased substrate stiffness significantly enhanced migrasome production, as evidenced by higher vesicle counts and elevated expression of migrasome-associated proteins in cell lysates (Fig. S9A and B). These results experimentally support the notion that increased microenvironment stiffness promotes migrasome formation by CAFs, providing mechanistic insight into how POSTN^+^ CAFs regulate migrasome biogenesis through biomechanical cues during liver cancer progression.

Beyond promoting tumor growth, POSTN^+^ CAFs also play a key role in immune evasion. We found that these cells, together with their secreted migrasomes, contribute to the formation of an “immune barrier” at the tumor edge. This barrier impedes immune cell infiltration and dampens immune surveillance, thereby fostering resistance to ICB therapies. The physical and immunosuppressive characteristics of this barrier represent a major obstacle to effective immunotherapy in liver cancer. Thus, POSTN^+^ CAFs and their migrasomes not only sustain tumor growth but also help the tumor evade immune destruction, posing a dual threat to therapeutic efficacy.

While previous studies—such as that by Wang et al. [21]—have highlighted the physical barrier formed by interactions between POSTN^+^ CAFs and SPP1^+^ macrophages, our findings provide a complementary perspective by emphasizing the role of migrasomes in immune resistance. We demonstrate that migrasomes derived from POSTN^+^ CAFs are strongly associated with poor outcomes in immunotherapy-treated patients, not only in liver cancer but also in bladder cancer, melanoma, and metastatic urothelial carcinoma. These migrasomes, concentrated at the tumor boundary, likely contribute to a migrasome-mediated immune blockade that limits T cell access to the tumor. While the precise mechanisms remain to be fully elucidated, our findings suggest that migrasomes may participate in or even reinforce the physical barrier identified by Wang et al., thereby representing an additional mechanism of immune evasion.

Although this study primarily focuses on the role of CAF-derived migrasomes in HCC, it offers broader insights into tumor–stroma interactions and unveils potential therapeutic strategies targeting the stromal compartment. Our findings underscore 3 major functional roles of CAF-derived migrasomes in liver cancer progression: (a) promoting angiogenesis within the tumor core by delivering pro-angiogenic cargo such as VEGFA, thereby sustaining tumor growth and vascularization; (b) reprogramming neighboring nonmalignant hepatocytes via enhanced oxidative phosphorylation and ROS accumulation, which induces early malignant transformation; and (c) cooperating with POSTN^+^ CAFs to form a physical and biochemical immune barrier at the tumor–stroma interface that impedes immune cell infiltration and diminishes the efficacy of ICB therapy.

**Fig. 8.**
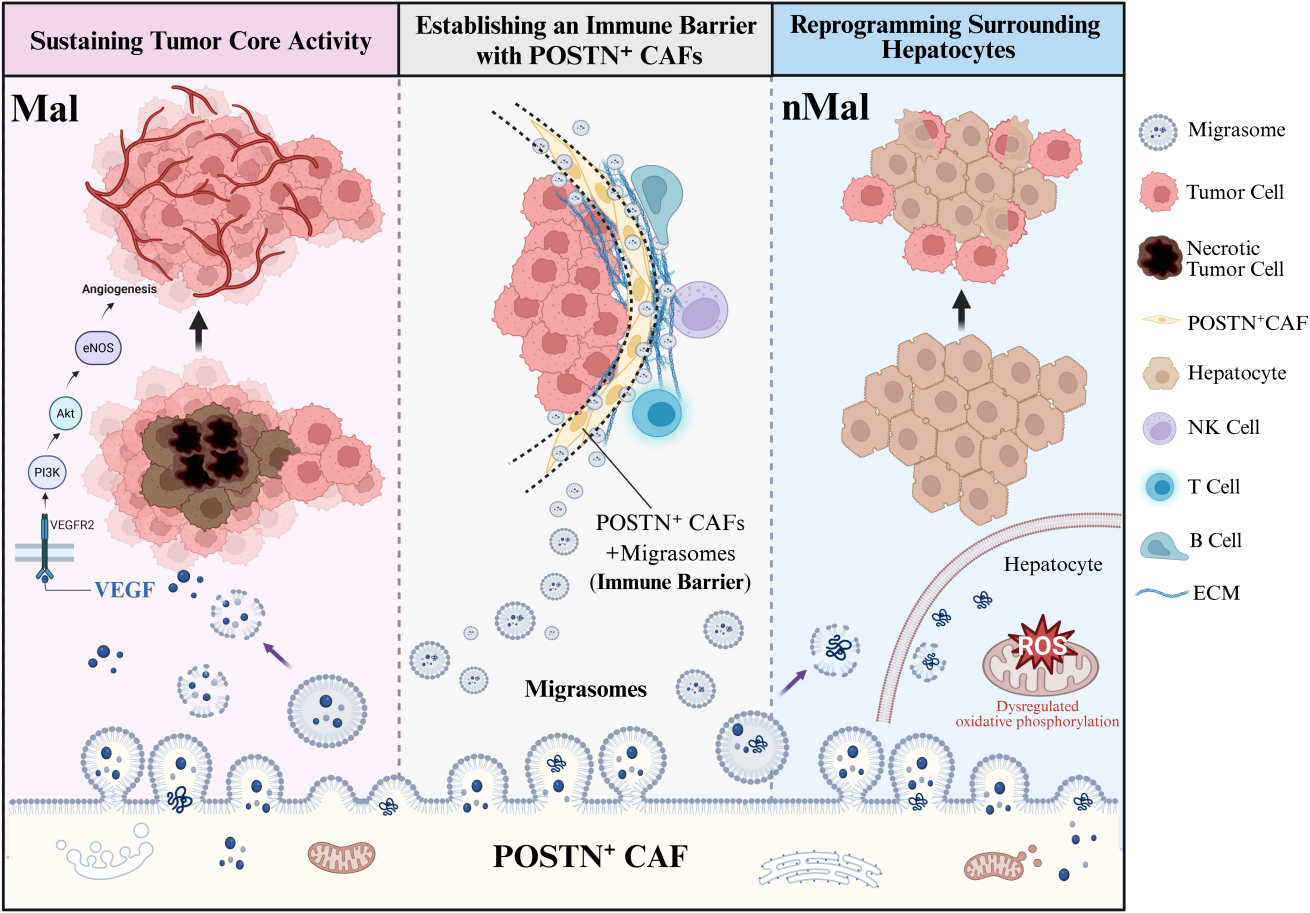
CAF-derived migrasomes, particularly from the POSTN+CAF subpopulation, play a critical role in liver cancer progression. These migrasomes are primarily concentrated at the tumor boundary, where they enhance the proliferative and invasive capabilities of liver cancer cells. Moreover, migrasomes can spread into adjacent nonmalignant liver cells, gradually inducing their malignant transformation. Additionally, POSTN+CAF-derived migrasomes form a barrier that impedes immune checkpoint inhibitor (ICB) efficacy, promoting immune evasion.

Targeting POSTN^+^ CAFs or disrupting the structural and metabolic functions of migrasomes may help dismantle this immune-evasive niche and improve responsiveness to immunotherapies. Furthermore, therapeutic strategies aimed at inhibiting migrasome biogenesis or blocking their paracrine signaling could simultaneously suppress angiogenesis, limit tumor spread, and prevent the malignant reprogramming of adjacent normal tissue (Fig. 8). These findings position CAF-derived migrasomes as promising therapeutic targets and offer a conceptual framework for stroma-targeted interventions in liver cancer and potentially other solid tumors.

## Methods

### Data collection

Bulk RNA-seq data were primarily used to compare the levels of migrasomes across different stages and grades of liver cancer, as well as to assess the differential levels of migrasomes between immunotherapy responders and nonresponders. Data from the IMvigor210 cohort [53], GSE91061, and GSE176307 were utilized for these analyses. For single-cell transcriptomic analysis, datasets from the Gene Expression Omnibus (GEO), including GSE125449, GSE156337, GSE98638, CNSA (CNP0000650), and EGA (EGAS00001003449), were processed. Normal samples were excluded, and only HCC tumor-derived single-cell data were retained, while non-HCC samples such as cholangiocarcinoma (CCA) were excluded, to compare migrasome-related features across various cell types. The EGA data for this study are available under accession number EGAS00001003449. Spatial transcriptome sequencing data of 2 HCC tumor samples were downloaded from Pmid35673582. To explore spatial gene expression patterns, we retrieved tumor-section spatial transcriptome data from 8 HCC patients (including 5 who did not respond and 3 who responded to anti-PD-1 therapy) based on the dataset provided by Liu et al [54].

### Dimension reduction and clustering

To process the 4 HCC single-cell transcriptome datasets, we utilized the Seurat package. Initially, variable genes were selected using the FindVariableFeatures function, and data normalization was performed. To mitigate intersample batch effects, we applied the RunHarmony function from the harmony package [55]. Clusters were identified via Seurat’s FindClusters function under multiple resolutions. For visualization and dimensionality reduction, UMAP was employed through RunUMAP with the following settings: reduction = “harmony” and dims = 1:10.

### Inference of malignant tumor cells

To discriminate malignant from normal cells in liver cancer datasets, we adopted the CopyKAT tool (Copy number Karyotyping of Aneuploid Tumors), an R-based algorithm for detecting large-scale copy number variations (CNVs) from single-cell RNA-seq profiles [29]. Each HCC sample was processed separately using normalized gene expression matrices. All parameters were maintained at default settings. CNV-based classification results were visualized and served as the foundation for distinguishing tumor from nontumor cells.

### Migrasome_Score calculation

We calculated the Migrasomes Score via GSVA [56], using a 7-gene signature linked to migrasome function. Scoring at the single-cell level followed previously published workflows [57] (https://www.github.com/cssmillie/ulcerative_colitis). For each cell, the score was computed as the mean of scaled expression values for all genes in the signature.

### Spatial transcriptomic tumor boundary definition

Using the Cottrazm package, tumor regions (malignant core, boundary zone, and adjacent nonmalignant tissue) on spatial transcriptomics slides were annotated via the BoundaryDefine function [58]. The find_neighbors function was employed to outline the immediate neighboring zones inside and outside the tumor margin.

### Spatial spot deconvolution

To determine the cell-type composition in each spatial transcriptomics (ST) spot, we utilized the SpatialDecon function. Visualization of spot-level cellular proportions was performed with DeconPieplot, both available within the Cottrazm package.

### Analysis of intercellular communication

Cell–cell signaling pathways were investigated using the CellChat R package (v1.5.0), incorporating curated ligand–receptor interaction databases such as CellChatDB and Omnipath [59,60]. Communication probabilities between cell types were estimated based on average ligand expression in sender clusters and receptor expression in receiver clusters. Significance was evaluated through permutation testing. Key interactions were visualized using the netVisual_circle, netVisual_heatmap, and netVisual_bubble functions.

### Survival analysis

To stratify patients based on migrasome activity, the optimal threshold separating high and low Migrasome_Score groups was computed using the maxstat.test function in the maxstat package. Kaplan–Meier curves and log-rank tests were used to evaluate survival differences between these groups.

### DEG identification and functional enrichment

Marker genes distinguishing different CAF subtypes were identified with the FindAllMarkers function in Seurat, applying min.pct = 0.2, logfc.threshold = 0.25, and only.pos = TRUE. Differential expression was assessed via the Wilcoxon rank-sum test, with Bonferroni-adjusted *P* values. Gene expression was log-transformed and scaled for heatmap visualization. Functional enrichment of the identified DEGs was conducted using clusterProfiler (v4.2.2), applying a hypergeometric test for statistical significance.

### Tissue preparation

HCC tumor tissues along with paired adjacent normal samples were collected from Shanghai General Hospital. The study protocol received approval from the hospital’s ethics committee. All participants provided informed consent. Clinical data are detailed in Table S2.

### Cell lines and animal models

The human HCC cell lines MHCC97-L and Hep3B were acquired from Zhejiang Meisen Cell Technology Co. Ltd., while the mouse hepatocyte lines NCTC-1469 and AML12 were sourced from the American Type Culture Collection (ATCC) (USA). All cell lines underwent contamination screening prior to experimentation.

For the xenograft study, 1 × 10^6^ cells were injected subcutaneously into 7-week-old BALB/c nu/nu mice (Shanghai Institute of Physical Medicine, Chinese Academy of Sciences). Tumor growth was monitored weekly.

For subcutaneous xenograft assays, 1 × 10^6^ cells were implanted into the flanks of male BALB/c nude mice (7 weeks old, Shanghai Institute of Physical Medicine). Tumor dimensions were recorded on a weekly basis.

In orthotopic tumor models, 2 strategies were employed: (a) Tumors pre-established subcutaneously were surgically transferred into the liver of nude mice, and (b) 1 × 10^6^ cells were injected directly into the liver parenchyma. Mice were allowed to progress tumor development naturally before being sacrificed for tissue collection.

In vivo tumorigenicity assay: To evaluate whether CAF-derived migrasomes can endow normal hepatocytes with tumorigenic potential, we performed subcutaneous injection experiments in immunodeficient mice. Briefly, AML12 cells were treated under 3 conditions: Control group: AML12 cells treated with PBS (negative control); Mig1 group: AML12 cells pre-incubated with CAF-derived migrasomes; Mig2 group: AML12 cells pre-incubated with CAF-derived migrasomes as above, followed by a second intradermal injection of purified migrasomes near the injection site on day 2 post-implantation. In each experimental group, 1 × 10^6^ AML12 cells suspended in 100 μl of PBS were implanted subcutaneously into the flanks of 7-week-old male BALB/c nude mice. The animals were observed daily for signs of tumor emergence, localized inflammation, or regression.

All animal experiments were conducted in accordance with institutional ethical standards approved by the Animal Ethics Committee of Shanghai Tongren Hospital.

### Western blot

Whole-cell lysates were prepared using the KeyGen Total Protein Extraction Kit, and protein concentrations were quantified using the bicinchoninic acid (BCA) method (KeyGen BioTECH, China). Proteins were resolved via 10% sodium dodecyl sulfate–polyacrylamide gel electrophoresis (SDS-PAGE) and transferred onto polyvinylidene difluoride (PVDF) membranes. Primary antibodies targeting integrin α5, β-actin, EOGT, TSPAN7, PIGK, TSPAN4, CD9, and CD63 were applied at 1:1,000 dilution, followed by horseradish peroxidase (HRP)-conjugated secondary antibodies. Protein bands were visualized using enhanced chemiluminescence.

### Confocal microscopy

Cells were cultured in confocal dishes for approximately 12 h. After fixation in 4% paraformaldehyde, samples were labeled with WGA488 dye (1 μg/ml for cells, 2 μg/ml for tissues). Confocal images were captured using a NIKON A1RSiHD25 system at a resolution of 1,024 × 1,024 pixels.

### Migrasome isolation

Migrasomes were separated from cell culture media using OptiPrep-based iodixanol–sucrose gradient ultracentrifugation (Sigma-Aldrich). The multi-step process involved centrifugation at 1,000*g* (5 min), 4,000*g* (20 min), and 20,000*g* (20 min) at 4 °C to eliminate cells and debris. The resulting pellet was resuspended and subjected to density gradient separation at 150,000*g* for 4 h. Fractions ranging from 3% to 27% iodixanol were collected and purified through additional washes and centrifugations.

### Transmission electron microscopy

Cells were initially fixed with a 1:1 mix of culture medium and 2.5% glutaraldehyde for 5 min, then postfixed in glutaraldehyde (2 h). After PBS washes, samples were dehydrated through graded ethanol and embedded in SPON12 resin. Ultrathin 70-nm sections were cut, mounted on copper grids, and stained with uranyl acetate and lead citrate. Imaging was carried out using a Hitachi H-7650B transmission electron microscope at 80 kV.

### Transwell invasion assay

Invasion capacity was evaluated using Matrigel-coated Transwell inserts (8-μm pores, Corning). Cells (1.5 × 10^5^) suspended in serum-free medium were placed in the upper chamber, while 10% fetal bovine serum (FBS)-containing medium served as a chemoattractant in the lower chamber. After 48 h, noninvasive cells were removed and invasive cells were stained with crystal violet and quantified.

### Scratch wound migration assay

Monolayers of cells grown in 6-well plates were wounded using sterile pipette tips. Detached cells were washed away, and wound closure was documented at 0 h and at later time points. ImageJ was employed to measure the scratch width and assess migration.

### Field emission SEM

Samples were prefixed in 2.5% glutaraldehyde at 4 °C and postfixed with 1% osmium tetroxide plus 1.5% potassium ferrocyanide. After ethanol dehydration, samples underwent tert-butanol substitution and were freeze-dried. A 10-nm gold layer was sputter-coated, and imaging was conducted on a field emission SEM at 3 kV.

### Angiogenesis assay

HUVECs were synchronized in low-serum ECM and then seeded (5 × 10^4^ cells per well) onto presolidified Matrigel (BD Biosciences) in 96-well plates. After 4 h at 37 °C, angiogenic structures were imaged using a standard light microscope and quantified.

### TMA and immunohistochemistry

TMAs containing paired tumor and peritumoral samples (*n* = 90 pairs) were prepared by Shanghai Zhuoli Biotech. Slides were deparaffinized, rehydrated, and blocked with 10% normal goat serum. Primary antibody against TSPAN4 (Abcam) was incubated overnight at 4 °C. After HRP-conjugated secondary antibody staining, diaminobenzidine (DAB) chromogen was applied. Slides were counterstained and mounted for histological evaluation.

### Seahorse metabolic profiling

Cellular respiration [oxygen consumption rate (OCR)] and glycolysis [extracellular acidification rate (ECAR)] were measured using the XF96 Analyzer (Seahorse Bioscience). Cells were plated in nonbuffered RPMI media supplemented with glucose, glutamine, and pyruvate. Mitochondrial inhibitors—oligomycin, carbonyl cyanide-p-trifluoromethoxyphenylhydrazone (FCCP), rotenone, and antimycin A—were sequentially injected. Data analysis was performed using Seahorse Wave software (v2.6.1.53) [61].

### Flow cytometry

Single-cell suspensions were prepared from freshly isolated tumor tissues. Briefly, tumors were mechanically dissociated and enzymatically digested with collagenase and deoxyribonuclease (DNase) I, followed by filtration through a 70-μm cell strainer. After red blood cell lysis, cells were washed and resuspended in fluorescence-activated cell sorting (FACS) buffer (PBS with 2% FBS). Dead cells were excluded using the Live/Dead Fixable Aqua dye (BD, catalog no. 564406). Cells were then incubated with the following fluorochrome-conjugated monoclonal antibodies for 30 min at 4 °C in the dark: CD45 (BD, catalog no. 557659), CD3 (BD, catalog no. 553061), CD8 (BD, catalog no. 566985), PD-1 (BD, catalog no. 562671), and TIM-3 (BD, catalog no. 566346). After washing, samples were acquired on a BD LSRFortessa flow cytometer, and data were analyzed using FlowJo software (TreeStar, v10). The gating strategy was as follows: live CD45^+^ leukocytes, CD3^+^ T cells, CD8^+^ cytotoxic T cells, and PD-1 and TIM-3 expression. The frequency of PD-1^+^TIM-3^+^ double-positive CD8^+^ T cells was quantified and compared between groups.

### Statistical analyses

All experiments were conducted in triplicate unless stated otherwise. Data were expressed as mean ± SD. Statistical significance was tested using unpaired *t* tests or one-way analysis of variance (ANOVA) followed by Tukey’s post hoc correction via GraphPad Prism 6.0. A significance level of *P* < 0.05 was considered statistically significant (*P < 0.05, **P < 0.01, and ***P < 0.001).

## Data Availability

The datasets analyzed in this study are publicly accessible from open repositories, including TCGA, ICGC, GEO, and EGA. Additional data supporting the findings of this study are available from the corresponding author upon reasonable request.
