# Supplementary material for: POSTN+ CAF-Derived Migrasomes Drive Hepatocellular Carcinoma Progression and Confer Resistance to Immunotherapy
Source: Research (Wash D C). 2025 Oct 22;8:0950. doi: 10.34133/research.0950 (PMC12541147; doi:10.34133/research.0950)
Supplement: Supplementary 1 — Tables S1 to S3 Figs. S1 to S9 [file research.0950.f1.zip › Supplementary Figures.docx]

**Supplementary Figures
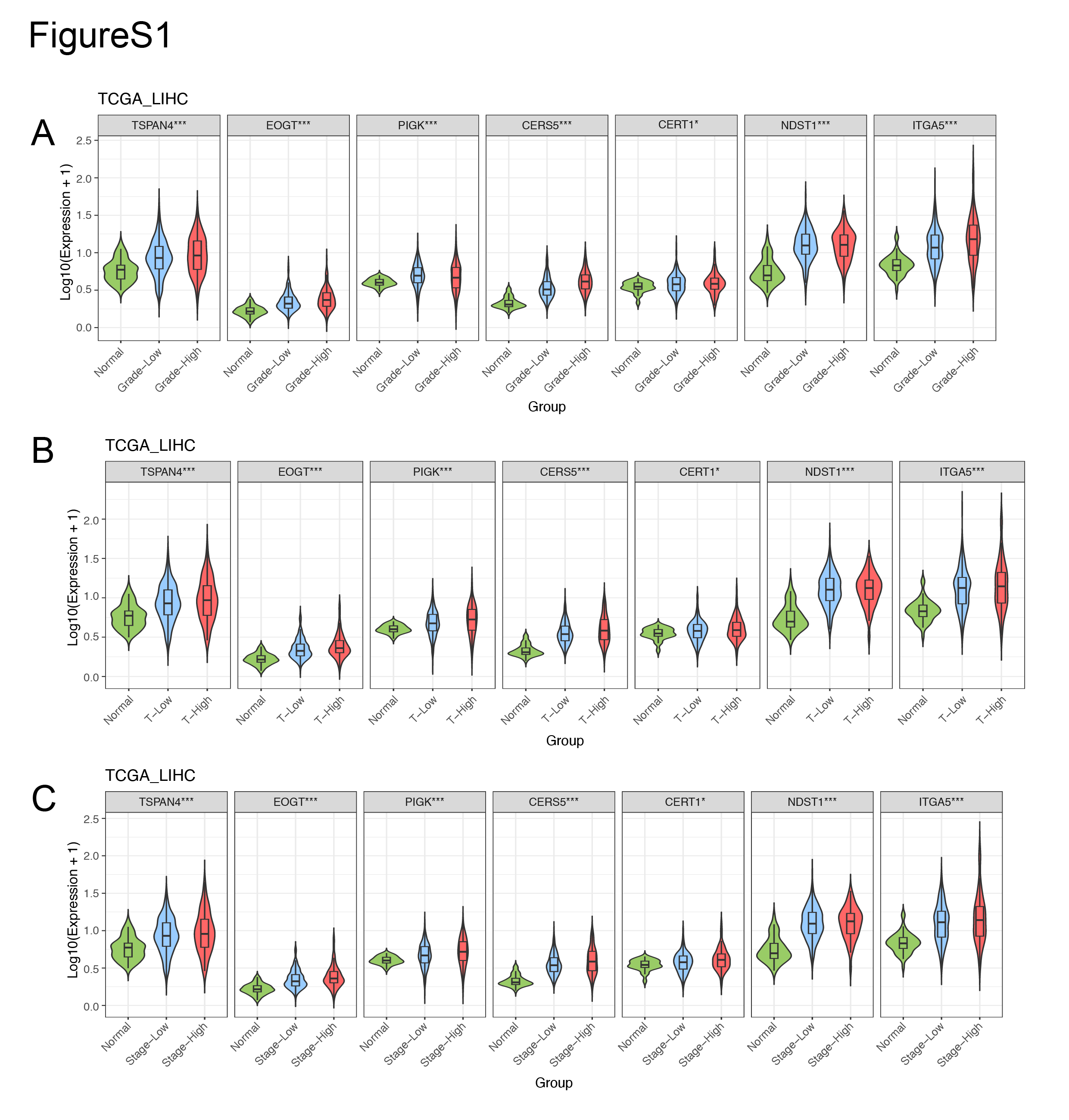
**

**Figure S1: Expression analysis of migrasome-related genes in different stages of liver cancer in the TCGA dataset.**
A: Box plots showing the expression of migrasome-related genes across different Grades in liver cancer. B: Box plots showing the expression of migrasome-related genes across different T stages in liver cancer. C: Box plots showing the expression of migrasome-related genes across different stages in liver cancer. Data information: Data are expressed as mean ± SD. Two-tailed unpaired Student's t-tests. One-way analysis of variance (ANOVA) was used followed by Tukey's post hoc test to determine the statistical significance, **p < 0.05; **p < 0.01; ***p < 0.001; ns, not significant.*


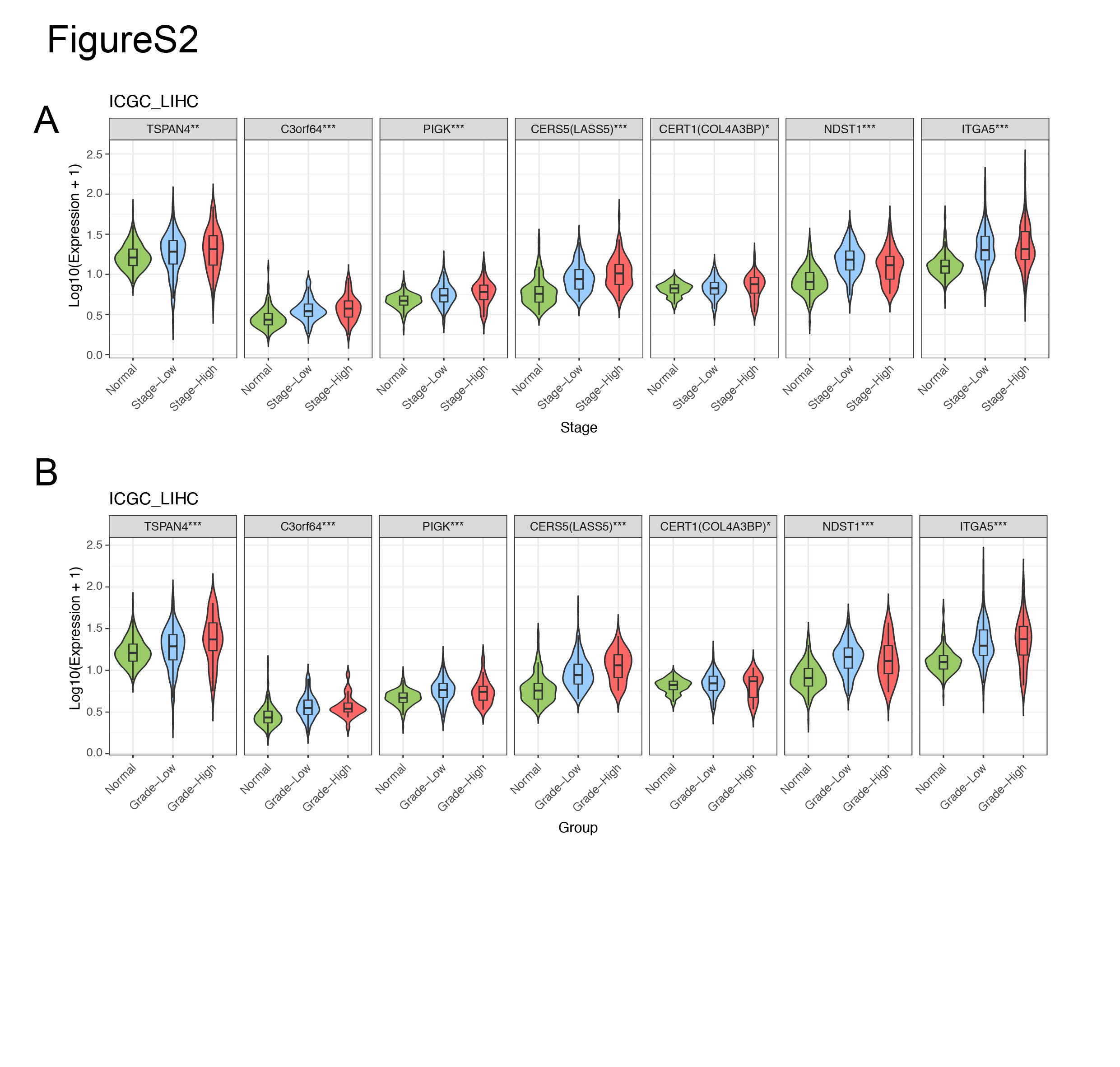


**Figure S2: Expression analysis of migrasome-related genes in different stages of liver cancer in the ICGC dataset.**
A: Box plots showing the expression of migrasome-related genes across different stages in liver cancer. B: Box plots showing the expression of migrasome-related genes across different Grades in liver cancer. Data information: Data are expressed as mean ± SD. Two-tailed unpaired Student's t-tests. One-way analysis of variance (ANOVA) was used followed by Tukey's post hoc test to determine the statistical significance, **p < 0.05; **p < 0.01; ***p < 0.001; ns, not significant.*


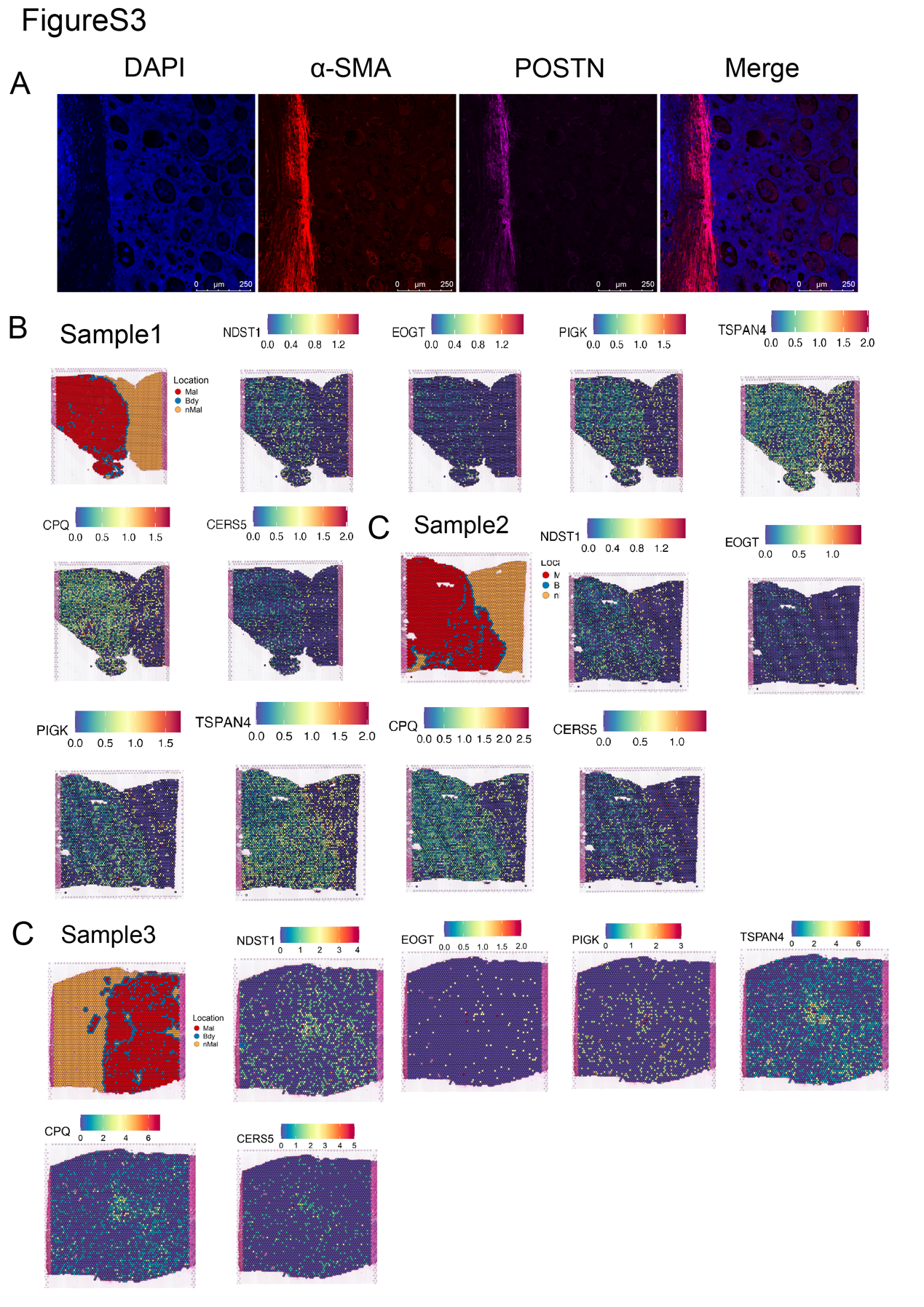


**Figure S3: Spatial sample analysis of liver cancer tissues.**
A: Immunofluorescence staining of human liver cancer samples, with red representing α-SMA (CAF marker), magenta representing POSTN (POSTN^+^CAF marker), and blue representing DAPI (cell nuclei) (Scale bars: 250µm). B-D: Expression and localization of single migrasome marker genes in liver cancer spatial transcriptomic samples.


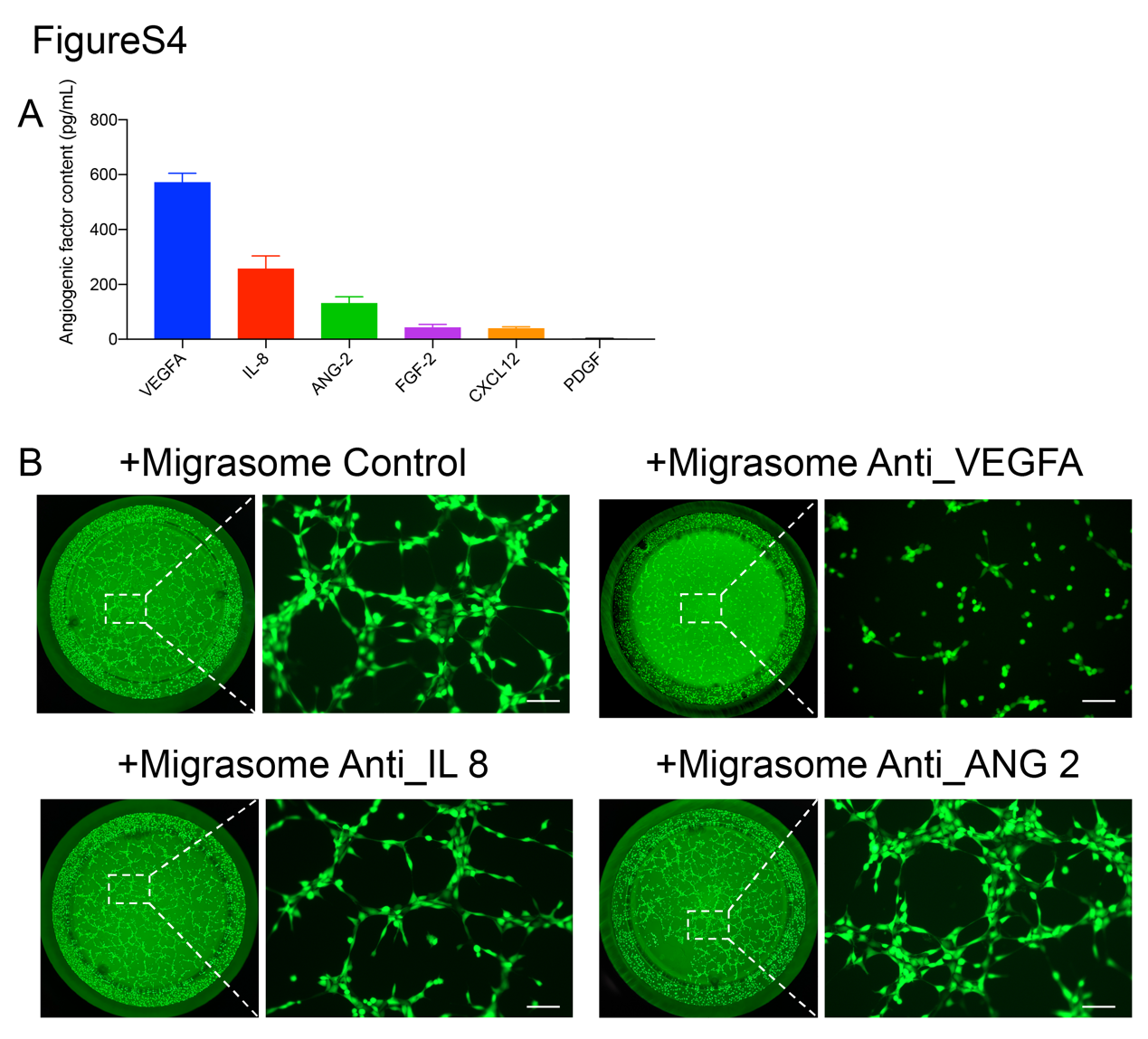


**Figure S4: Quantification of angiogenic factors in CAF-derived migrasomes and blocking experiments in HUVECs.**
A: ELISA quantification of multiple angiogenic factors, including VEGFA, IL-8, ANG-2, FGF-2, CXCL12, and PDGF, in CAF-derived migrasomes. VEGFA exhibited the highest concentration among all measured factors. Data are presented as mean ± SD from three independent experiments. B: Tube formation assay of HUVECs treated with CAF-derived migrasomes in the presence or absence of receptor-specific inhibitors targeting VEGFR (for VEGFA), CXCR1/2 (for IL-8), or Tie2 (for ANG-2). Representative images and quantification of tube length are shown. Scale bars, 100 µm.


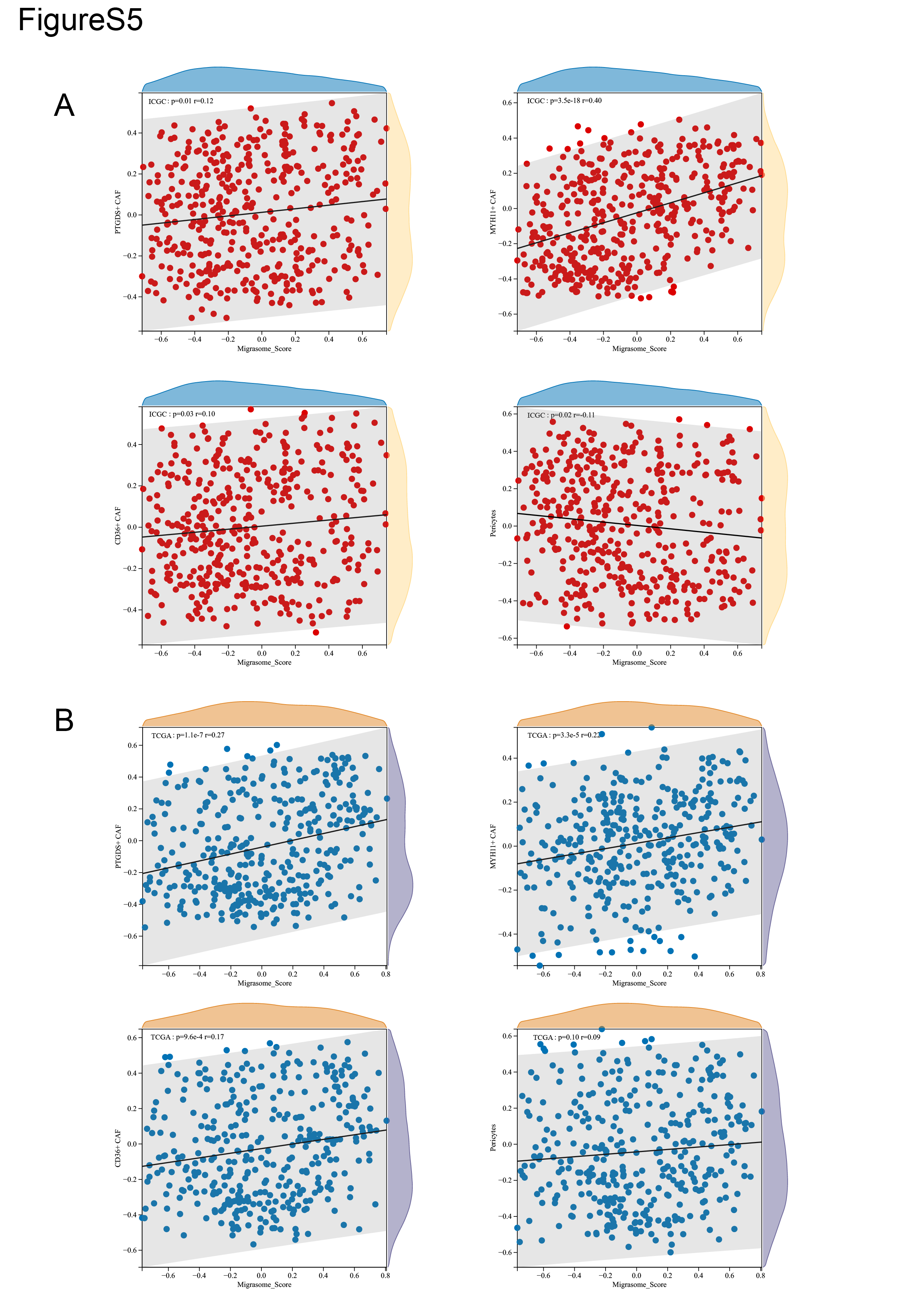


**Figure S5: Correlation analysis of different CAF subtypes and Migrasome-related genes in TCGA and ICGC datasets.**
A: Correlation analysis showing the relationship between different CAF subtypes and Migrasome_Score in ICGC-LIRI samples. B: Correlation analysis showing the relationship between different CAF subtypes and Migrasome_Score in TCGA-LIHC samples.

**
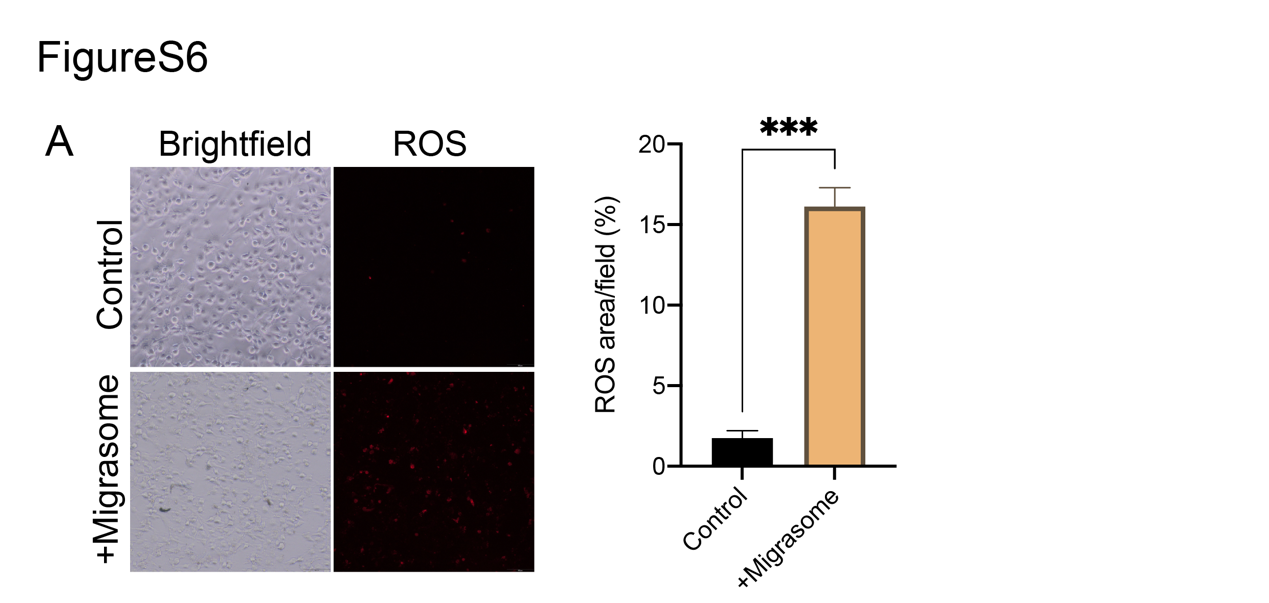
**

**Figure S6: Effects of Migrasomes on Mitochondrial Metabolism in Cells**

A: Bright-field and ROS immunofluorescence staining in AML12 (Control) and AML12 + Migrasome (+Migrasome) cells. Data information: Data are expressed as mean ± SD. Two-tailed unpaired Student's t-tests, **p < 0.05; **p < 0.01; ***p < 0.001; ns, not significant.*


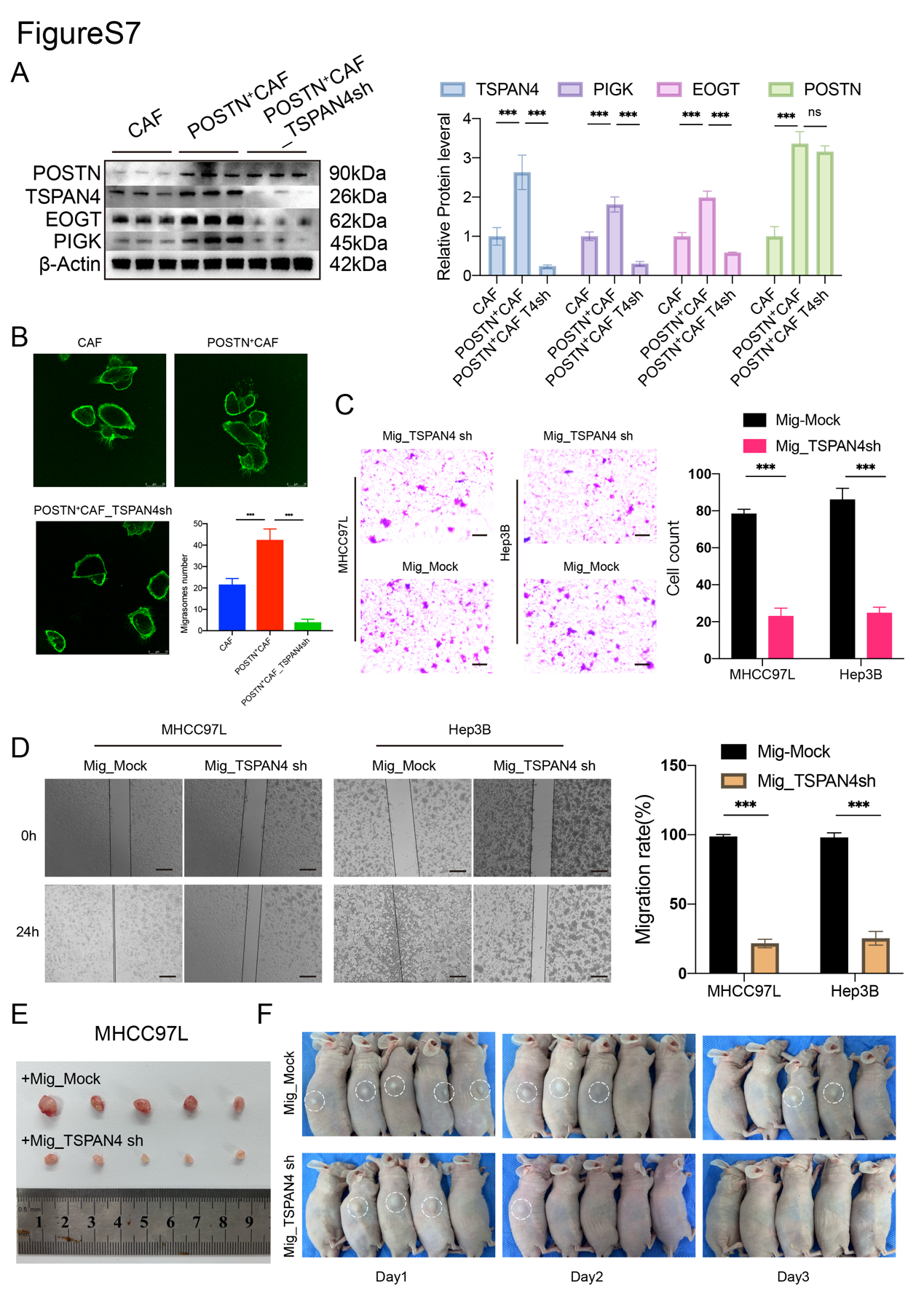


**Figure S7: POSTN⁺ CAFs are major producers of migrasomes and promote liver cancer progression.**

A: Western blot analysis of migrasome-associated proteins in parental CAFs, POSTN⁺ CAFs, and POSTN⁺CAF_TSPAN4sh cells. POSTN⁺ CAFs exhibited elevated expression of migrasome markers, whereas TSPAN4 knockdown markedly reduced their levels. B: Representative confocal immunofluorescence images showing migrasome formation in POSTN⁺ CAFs and POSTN⁺CAF_TSPAN4sh cells. C–D: Transwell invasion (C) and migration (D) assays of MHCC97L and Hep3B cells treated with migrasomes derived from POSTN⁺CAF_Mock or POSTN⁺CAF_TSPAN4sh cells. Migrasomes from POSTN⁺CAF_Mock significantly enhanced invasive and migratory capacities compared with TSPAN4-silenced controls. E: Representative images and quantification of subcutaneous tumors formed by MHCC97L cells pretreated with migrasomes from POSTN⁺CAF_Mock or POSTN⁺CAF_TSPAN4sh cells. Migrasomes from POSTN⁺CAF_Mock promoted larger tumor growth in vivo. F: In vivo tumorigenicity assay using normal liver cells pretreated with migrasomes from POSTN⁺CAF_Mock or POSTN⁺CAF_TSPAN4sh cells. POSTN⁺CAF_Mock-derived migrasomes induced more frequent and persistent tumor formation, whereas POSTN⁺CAF_TSPAN4sh-derived migrasomes showed reduced tumorigenic capacity. Data information: Data are expressed as mean ± SD. Two-tailed unpaired Student's t-tests, **p < 0.05; **p < 0.01; ***p < 0.001; ns, not significant.*

**
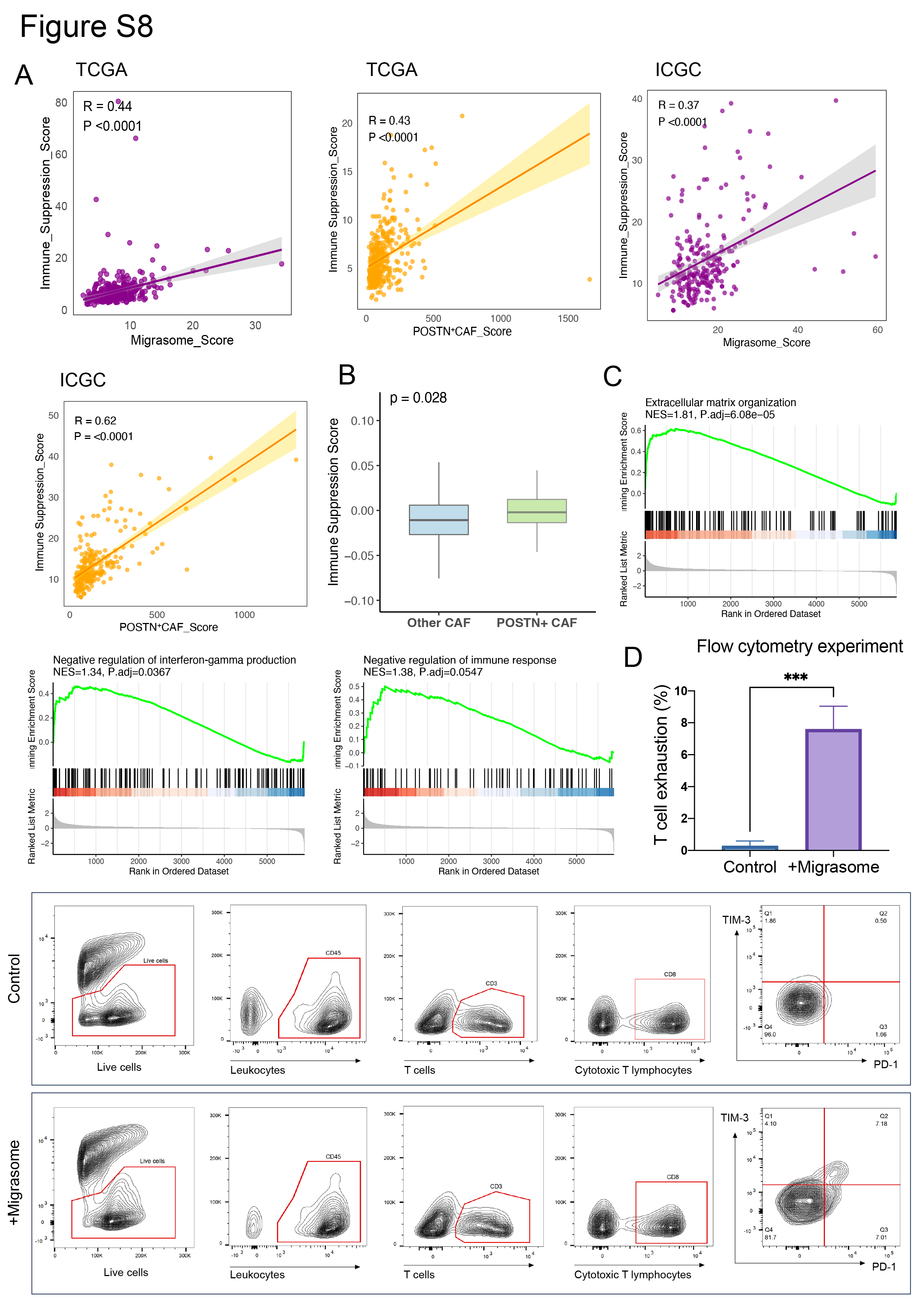
**

**Figure S8: Correlation of Migrasome_Score, Immune Suppression_Score, and POSTN⁺CAF_Score, and immunosuppressive features of POSTN⁺ CAFs.**

A: Correlation analyses between Migrasome_Score and Immune Suppression_Score, as well as between POSTN⁺CAF_Score and Immune Suppression_Score, in TCGA and ICGC liver cancer cohorts. B: Comparison of immune suppression scores among CAF subpopulations at the single-cell level, showing higher scores in POSTN⁺ CAFs relative to other CAF subsets. C: Gene Set Enrichment Analysis (GSEA) indicating that POSTN⁺ CAFs are significantly enriched in multiple immune-suppressive and extracellular matrix (ECM)-related pathways. D: Representative flow cytometry plots and quantitative analysis showing the proportion of PD-1⁺TIM-3⁺ exhausted cells within CD8⁺ T cells isolated from tumor tissues of mice treated with migrasomes or control. Data information: Data are expressed as mean ± SD. Two-tailed unpaired Student's t-tests, **p < 0.05; **p < 0.01; ***p < 0.001; ns, not significant.*


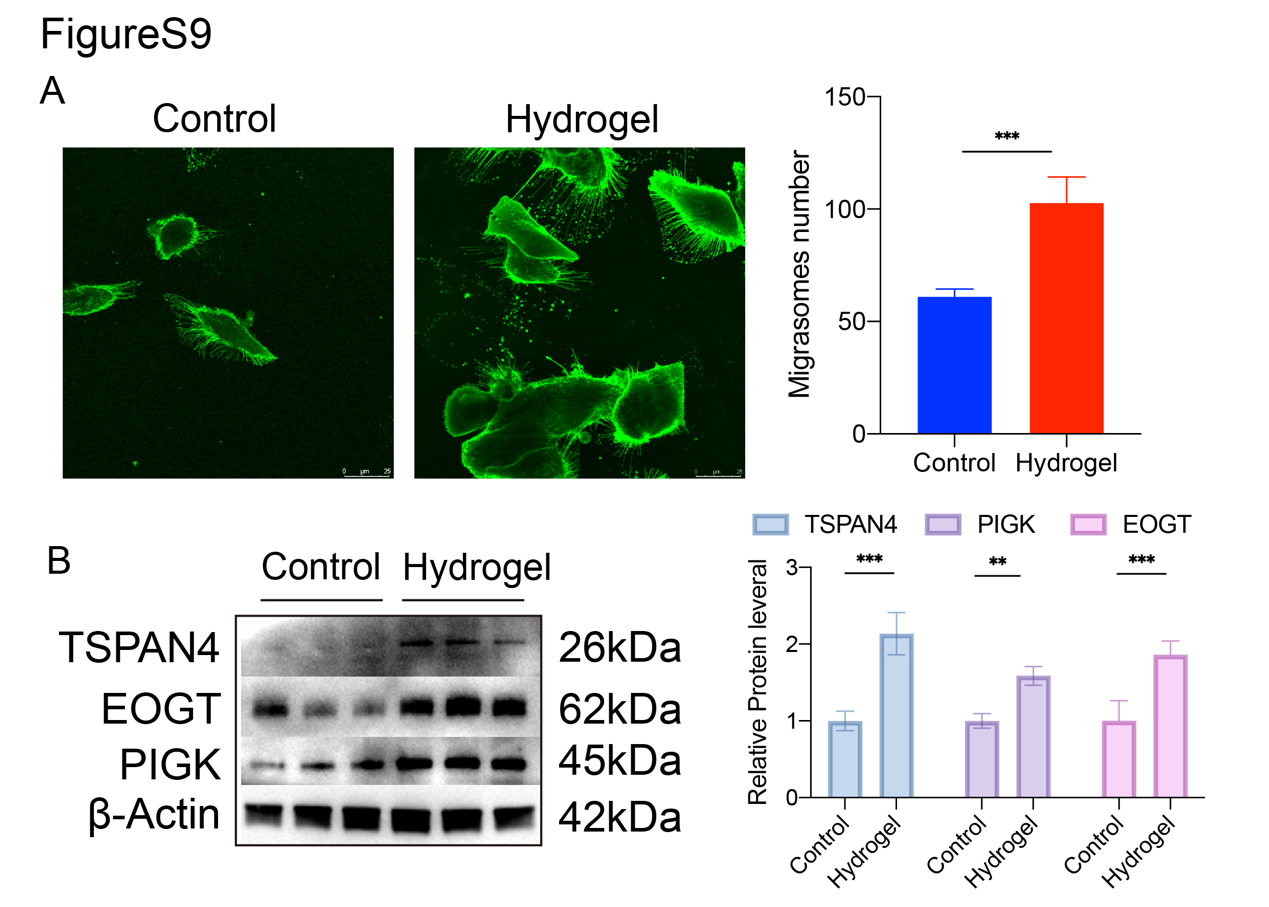


**Figure S9: Increased migrasome production by CAFs cultured on hydrogel substrates.**

A: Representative confocal microscopy images showing migrasome formation by CAFs cultured under standard conditions (normal culture) and on VitroGel hydrogel substrates.

B: Western blot analysis of migrasome-associated protein expression in CAFs cultured under normal conditions or on hydrogel substrates. Increased expression of migrasome markers was observed in hydrogel-cultured CAFs compared to controls. Data information: Data are expressed as mean ± SD. Two-tailed unpaired Student's t-tests, **p < 0.05; **p < 0.01; ***p < 0.001; ns, not significant.*
